# Application of Machine Learning in the Development of Fourth Degree Quantitative Structure–Activity Relationship Model for Triclosan Analogs Tested against *Plasmodium falciparum* 3D7

**DOI:** 10.1021/acsomega.4c05768

**Published:** 2024-10-25

**Authors:** Railton Marques de Souza Guimarães, Ivo Henrique Provensi Vieira, Fabrício Berton Zanchi, Rafael Andrade Caceres, Fernando Berton Zanchi

**Affiliations:** † Laboratório de Bioinformática e Química Medicinal, Fundação Oswaldo Cruz Rondônia, Porto Velho, Rondônia 76812-245, Brazil; ‡ Faculdade de Biomedicina, Centro Universitário Afya, Porto Velho, Rondônia 76805-846, Brazil; § Centro de Formação em Ciências Ambientais-CFCAm da Universidade Federal do Sul da Bahia-UFSB, Porto Seguro, Bahia 45810-000, Brazil; ∥ Laboratório de Bioinformática Estrutural, Modelagem Molecular e Simulação de Biossistemas—Programa de Pós-Graduação em Biociências—Universidade Federal de Ciências da Saúde de Porto Alegre—UFCSPA, Porto Alegre, Rio Grande do Sul 90050-170, Brazil; ⊥ Programa de Pós-Graduação em Biologia Experimental (PGBIOEXP), FIOCRUZ Rondônia/Universidade Federal de Rondônia (UNIR), Porto Velho, Rondônia 76812-245, Brazil; # Programa de Pós-Graduação Rede BIONORTE, FIOCRUZ Rondônia, Porto Velho, Rondônia 76812-245, Brazil; ¶ Instituto Nacional de Epidemiologia na Amazônia Ocidental—EPIAMO, Porto Velho, Rondônia 76812-245, Brazil

## Abstract

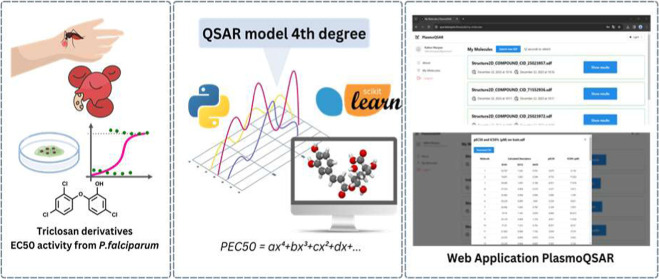

Despite new therapies against malaria, this disease remains one of the main causes of death affecting humanity. The phenomenon of resistance has caused concern as the drugs no longer have the same efficacy, forcing scientific research to develop new methods that prospect new molecules. With the advancement of artificial intelligence, it becomes possible to apply machine learning (ML) techniques in the discovery and evaluation of new molecules, by employing the quantitative structure–activity relationship (QSAR), a classic method that uses regressions to create a model that allows identifying and evaluating new drug candidates. This work combined QSAR with ML and developed a supervised model that modeled a fourth degree polynomial equation capable of identifying new drug candidates derived from the triclosan compound—a classic inhibitor of *Plasmodium falciparum* growth, the cause of severe malaria. The model produces an *R*^2^ greater than 80% for training and concurrent testing, as well as a correlation index greater than 80% between the calculated and experimental pEC_50_ (negative logarithm of half maximal effective concentration) data. In addition, a web software (PlasmoQSAR) was created that allows researchers to calculate the EC_50_ (half maximal effective concentration) of new molecules using the developed analytical method.

## 1 Introduction

Malaria is a serious and sometimes fatal disease caused by different types of parasites from the genus *Plasmodium*, such as *Plasmodium vivax*, *Plasmodium ovale*, *Plasmodium malariae*, and *Plasmodium falciparum*.^1^ Malaria is a life-threatening disease that affects nearly half of the world’s population, especially in tropical countries.^2^ According to the World Health Organization (WHO), there were an estimated 241 million cases of malaria and 627,000 deaths in 2020, mostly in Africa.^1^ Some countries in the Americas, such as Bolivia and Venezuela, also saw a significant rise in malaria cases, reaching more than 467,000 in 2019. However, it is very difficult to control, but there was a slightly decrease in 2020 cases, which could be related to the COVID-19 pandemic and other factors, such as reduced exposure to occupational risks,^3^ showing that, the existence of effective drugs and preventive measures, such as insecticide-treated bed nets, malaria remains a major challenge, especially in poor regions.^4^

Among the five *Plasmodium* species that cause malaria in humans, *P. falciparum* is the most deadly and prevalent in sub-Saharan Africa. *P. falciparum* can cause severe complications and high mortality rates if not treated promptly. Drug resistance is a serious problem that affects the treatment of malaria, a disease caused by *Plasmodium* parasites. Some of the most common and effective drugs, such as chloroquine and artemisinin, have lost their potency against some strains of *P. falciparum*, the most dangerous and widespread species of malaria parasite. This leads to more severe and fatal cases of malaria, especially in Africa. Therefore, it is very important to find and develop new drugs that can kill *P. falciparum* infections.^5,6^

Studies on the development of new antimalarial drugs have shown that the inhibition of fatty acid synthesis in *P. falciparum* is a promising alternative against drug-resistant strains.^7^ The synthesis of type II fatty acids (FAS-II) is essential for the survival of the parasite, directly participating in energy production and the synthesis of long-chain fatty acids, which are important components of the plasma membrane and the apicoplast, an organelle unique to apicomplexan protozoa.^8^ Among the enzymes of the cycle, we highlight the enoyl acyl reductase (PfFabl or PfENR) that catalyzes the last step of the cycle, reducing *trans*-2-enoil-ACP to acyl-ACP, dependent on reduced nicotinamide adenine dinucleotide.^9^ One of the most studied inhibitors against PfENR is triclosan. Since 2001, a study reported that triclosan, a common antibacterial agent, can inhibit the in vitro growth of *P. falciparum*, the most lethal malaria parasite. Since then, several studies have confirmed that triclosan and its derivatives can block the development of *P. falciparum* in the blood stage, which is responsible for the clinical symptoms of malaria.^10^

There is a lot of information about this enzymatic target, so it is common to find works applying various in silico techniques to identify new inhibitors of PfENR.^11−15^ We highlight here the quantitative structure–activity relationship (QSAR) that establishes a mathematical relationship between the chemical structure and the biological activity of a series of compounds, reducing the time and cost of developing new drugs, as it allows predicting the activity of new compounds without the need to synthesize or experimentally test them.^16^ To build a QSAR model, it is necessary to select a series of compounds with known activity, generate and choose the molecular descriptors that represent the properties of the compounds, establish an regression equation that relates the descriptors with the activity, and validate the model with statistical indicators and external data. QSAR can be used to predict the antimalarial activity of new candidates for FabI inhibitors,^12^ as well as to optimize their physicochemical and pharmacokinetic properties.^17^

Enhancing the knowledge of chemical hazards assessment, reduction of data dimensionality, optimization and validate the model parameters to understanding of new compounds,^18^ a combination of machine learning (ML) with QSAR models, can improve the quality and accuracy of the results.^19^

For this proposes, there are several tools, mathematical methodologies and libraries available to apply ML to QSAR, such as Scikit-learn,^20^ TensorFlow,^21^ PyTorch,^22^ RDKit,^23^ DeepChem,^24^ among others. These tools and libraries allow the implementation of different ML methods, such as linear regression, neural networks, support vector machines, decision trees, among others, to construct and evaluate QSAR models in an efficient and robust manner.^25^

In this work, we explore QSAR by applying ML, testing various descriptors in a polynomial model of multiple degrees in order to construct a mathematical model that calculates the pEC_50_ (negative logarithm of half maximal effective concentration) of triclosan derivatives against malaria disease. We also implemented the result of the polynomial equation that presented the best result in a WEB software called PlasmoQSAR, capable of calculating the half maximal effective concentration (EC_50_) of possible drug candidates against *P. falciparum*, the cause of malaria.

## 2 Materials and Methods

**Figure 1:**
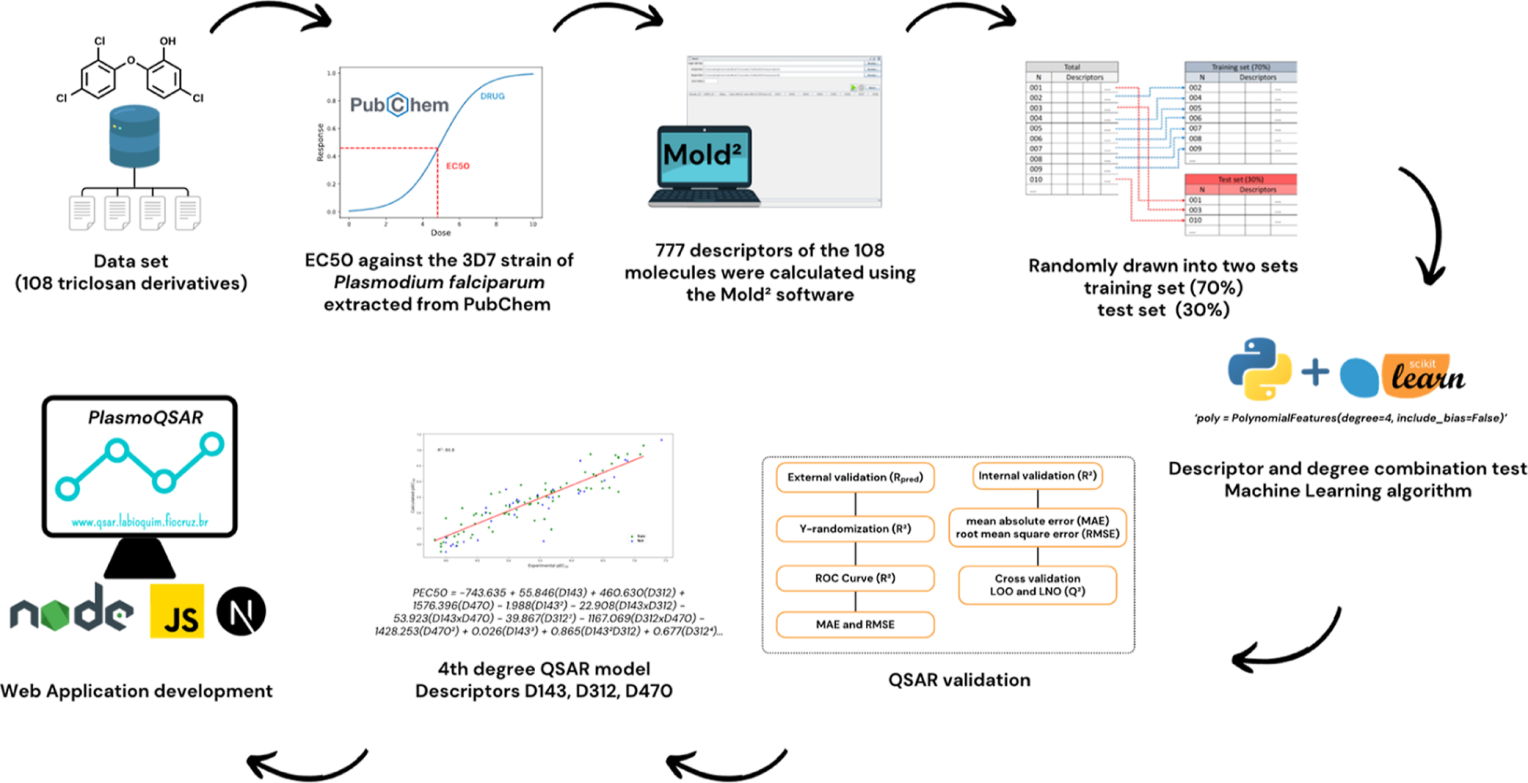
Flow of the stages of collection, construction and evaluation of the QSAR model as well as development of the WEB application.

From the collection, calculation of descriptors, separation of training and testing groups, construction of the program for combinations of descriptors and degrees, evaluation of QSAR models and implementation of the WEB software can be seen in Figure 1.

### 2.1 Data Set

A data set with 108 triclosan derivatives was taken from four works^26−29^ as shown in Table 1. To date, these are all the triclosan derivatives tested against *P. falciparum*. The EC_50_ of each molecule was extracted from PubChem against the 3D7 strain of *P. falciparum* and converted to the logarithmic scale pEC_50_ [=–log_10_(EC_50_)], also called the potency of the activity.

**Table 1:** Activity of Triclosan Derivatives against *P. falciparum* Strain 3D7 and Corresponding Work^a^

*N*	PubChem CID	EC_50_ (μM)	pEC_50_	active	work
**1**	5564	2.800	5.5528	Y	Anderson et al., 2013
**2**	44405339	100.000	4.0000	N	
**3**	71552938	0.081	7.0915	Y	
**4**	92602	52.000	4.2840	N	
**5**	71364024	38.000	4.4202	N	
**6**	71552773	4.700	5.3279	Y	
**7**	71552774	33.000	4.4815	N	
**8**	71552860	0.610	6.2147	Y	
**9**	71552937	0.130	6.8861	Y	
**10**	71552863	0.037	7.4318	Y	
**11**	71552864	0.130	6.8861	Y	
**12**	71552865	0.180	6.7447	Y	
**13**	71552935	0.072	7.1427	Y	
**14**	55224596	20.000	4.6990	N	
**15**	63461798	150.000	3.8239	N	
**16**	71552862	1.900	5.7212	Y	
**17**	71718729	9.700	5.0132	Y	
**18**	71552776	18.000	4.7447	N	
**19**	71552778	1.200	5.9208	Y	
**20**	627458	100.000	4.0000	N	
**21**	71552936	1.500	5.8239	Y	
**22**	71552939	0.200	6.6990	Y	
**23**	71552690	73.000	4.1367	N	
**24**	71552691	7.200	5.1427	Y	
**25**	71552861	2.000	5.6990	Y	
**26**	55193760	2.300	5.6383	Y	
**27**	71552777	0.730	6.1367	Y	
**28**	44405271	7.500	5.1249	Y	Freundlich et al., 2005
**29**	44405274	68.400	4.1649	N	
**30**	44405275	20.700	4.6840	N	
**31**	44405276	11.700	4.9318	N	
**32**	44405287	55.100	4.2588	N	
**33**	44405289	34.300	4.4647	N	
**34**	44405291	63.500	4.1972	N	
**35**	11674015	52.200	4.2823	N	
**36**	44405293	57.200	4.2426	N	
**37**	44405298	31.200	4.5058	N	
**38**	6914565	77.800	4.1090	N	
**39**	21272512	65.300	4.1851	N	
**40**	6852143	2.100	5.6778	Y	
**41**	6852148	120.000	3.9208	N	
**42**	11659169	3.900	5.4089	Y	
**43**	44405311	23.100	4.6364	N	
**44**	11660481	35.800	4.4461	N	
**45**	44405314	26.700	4.5735	N	
**46**	44405327	110.000	3.9586	N	
**47**	44405330	120.000	3.9208	N	
**48**	44405331	96.300	4.0164	N	
**49**	44405336	23.500	4.6289	N	
**50**	44405338	120.000	3.9208	N	
**51**	6914566	66.800	4.1752	N	
**52**	44405380	4.500	5.3468	Y	
**53**	44410066	0.250	6.6021	Y	Freundlich et al., 2006
**54**	44410081	0.320	6.4949	Y	
**55**	44410086	0.300	6.5229	Y	
**56**	11948630	0.140	6.8539	Y	
**57**	44410094	0.410	6.3872	Y	
**58**	44410097	3.500	5.4559	Y	
**59**	44410098	0.580	6.2366	Y	
**60**	44410128	0.450	6.3468	Y	
**61**	44410129	2.700	5.5686	Y	
**62**	44410130	0.770	6.1135	Y	
**63**	44410133	8.600	5.0655	Y	
**64**	44410134	0.180	6.7447	Y	
**65**	44410138	0.500	6.3010	Y	
**66**	44410170	11.000	4.9586	N	
**67**	44410210	0.230	6.6383	Y	
**68**	44410211	2.600	5.5850	Y	
**69**	44410215	2.100	5.6778	Y	
**70**	44410233	0.730	6.1367	Y	
**71**	44410234	14.000	4.8539	N	
**72**	44410238	0.630	6.2007	Y	
**73**	44410251	83.000	4.0809	N	
**74**	44410252	9.700	5.0132	Y	
**75**	44410256	0.320	6.4949	Y	
**76**	44410284	0.490	6.3098	Y	
**77**	44410286	0.330	6.4815	Y	
**78**	44410287	0.780	6.1079	Y	
**79**	44410291	0.210	6.6778	Y	
**80**	11495355	0.180	6.7447	Y	
**81**	44410295	19.000	4.7212	N	
**82**	23656593	100.000	4.0000	N	Freundlich et al., 2007
**83**	25023956	100.000	4.0000	N	
**84**	25023957	40.000	4.3979	N	
**85**	25023955	19.000	4.7212	N	
**86**	22947105	10.000	5.0000	Y	
**87**	25023954	2.300	5.6383	Y	
**88**	25023973	7.400	5.1308	Y	
**89**	16220130	7.500	5.1249	Y	
**90**	25023972	6.900	5.1612	Y	
**91**	25023971	11.000	4.9586	N	
**92**	16220129	8.100	5.0915	Y	
**93**	25023970	23.000	4.6383	N	
**94**	16220126	6.400	5.1938	Y	
**95**	25023969	11.000	4.9586	N	
**96**	16220128	5.600	5.2518	Y	
**97**	25023967	2.900	5.5376	Y	
**98**	25023966	3.500	5.4559	Y	
**99**	25023968	2.100	5.6778	Y	
**100**	25023964	3.400	5.4685	Y	
**101**	15942656	1.600	5.7959	Y	
**102**	25023965	2.600	5.5850	Y	
**103**	25023962	2.000	5.6990	Y	
**104**	25023963	7.400	5.1308	Y	
**105**	25023961	2.000	5.6990	Y	
**106**	25023959	2.500	5.6021	Y	
**107**	25023960	3.500	5.4559	Y	
**108**	25023958	4.600	5.3372	Y	

a The active correspond to active (Y) or not active (N).

### 2.2 Calculation and Selection of Descriptors

The descriptors were calculated using the free software Mold2 developed by the National Toxicology Center (NCTR), available in the Food and Drug Administration (FDA).^30^ The two-dimensional structure of all 108 molecules were retrieve from PubChem in SDF format and submitted to Mold2 in a single file, where 777 descriptors were calculated for each molecule. To select the best descriptors, a python program was developed using the ML library Scikit-learn.^31^ The program randomly splits the total set into two groups at each test: one for training and another for testing in the ratio 70:30, with 75 training and 33 test compounds. Then, at each iteration, it combines the descriptors into groups of three, executes the predictive machine training and calculates the coefficient of determination (*R*^2^) between the calculated pEC_50_ data set and experimental.

### 2.3 Multiple Regression Analysis

The multivariate method employed for regression analysis in QSAR model was the polynomial regression (PR). The algorithm tested the PR degrees ranging from 1 to 8 using the *PolynomialFeatures* class from Scikit-learn, adhering to a general model described by eq 1
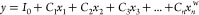
1

The variable *y* is the response (or dependent variable) that corresponds to pEC_50_, the coefficient *I*_0_ is the interception of *y*, *C*_1_, *C*_2_, *C*_3_ and *C*_*n*_ are the coefficients of the independent variables (descriptors) *x*_1_, *x*_2_, *x*_3_ and *x*_*n*_ respectively. As the degree of the polynomial increases, new terms are added to the equation, such as the interaction between descriptors that is, when the independent variables are multiplied by each other (*x*_1_*x*_2_, *x*_1_*x*_3_, *x*_2_*x*_3_, and so on).

### 2.4 QSAR Model Validation

The robustness and predictive power of a QSAR model depend on various validation methods applied to the training and test sets. The internal validation evaluates the predictive power of the training set, while the external validation is for the test set.

#### 2.4.1 Internal Validation

The goodness-of-fit of a QSAR model, indicated by *R*^2^, is evaluated by comparing the experimental and calculated data when the model is applied to the training data set (eq 2). The risk metrics mean absolute error (MAE) and root-mean-square error (RMSE) in eqs 3 and 4 respectively, were calculated to reinforce the reliability of the model’s predictions.
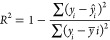
2
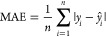
3
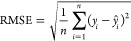
4In the equations above, the variable *y*_*i*_ represents the experimental or observed value, *n* is the number of compounds, *y̅*_*i*_ corresponds to the average of the observed activities and *ŷ*_*i*_ is the predicted activity of each observed value. The equations are imported from the sklearn library and calculated respectively by the *r2_score*, *mean_absolute_error* and *root_mean_squared_error* functions. The function *r2_score* scores the model on a scale between 0 and 1, where the closer to 1 the better the model’s performance. The combinations of descriptors that exhibit a satisfactory *R*^2^ value (*R*^2^ ≥ 0.6)^32^ of pEC_50_ for training are selected for external validations. The *mean_absolute_error* and *root_mean_squared_error* functions return the mean of the model error distribution. RMSE is a three-parameter function that penalizes minor errors, while MAE is a relatively simple function that penalizes large errors by averaging the absolute values of the errors. Therefore, it is considered a better indicator of errors in model performance analysis.^33^

#### 2.4.2 External Validation

External validation, also calculated by eq 2 (renamed to *R*_pred_^2^), 3 and 4, with the difference that the *r2_score*, *mean_absolute_error* and *root_mean_squared_error* functions is applied to the test data set, that is, those that did not participate in the training of the predictive machine.

#### 2.4.3 Cross-validation

The Leave-One-Out (LOO) and Leave-*N*-Out with *N* = 5 (LNO) cross-validation consists of excluding a LOO or five LNO molecules from the training set and reconstructing a new model from the remaining data. Then the cross-validated correlation coefficient *R*^2^ (named *Q*^2^) (eq 2) is calculated on the remaining data. The procedure was repeated until all possible sets were tested by removing a LOO or five LNO molecules. In the end, the average of the *Q*^2^ coefficients is calculated. It is advisible that the *R*^2^ value of the original model and *Q*^2^ is not greater than 30%^32^ to obtain a reliable prediction.

#### 2.4.4 Y-Randomization

The Y-randomization compares the performance of a model trained and applied to shuffled real data.^34^ With this, it is expected that the statistical parameters of the randomized model are smaller than the original. The set of responses was randomly randomized 100 times and the *R*^2^ (eq 2) of each randomization was calculated. Then the average of all randomizations is calculated.

#### 2.4.5 Correlation of Observed and Expected Values

A graph with all points representing the observed and expected pEC_50_ values are plotted. Then the regression line is calculated and plotted on the same graph with the *sns.regplot* functions from the seaborn library.^35^ The *R*^2^ of the correlation of the line with the plotted data is also calculated.

#### 2.4.6 ROC Curve for the Calculated Models

An effective way to measure the classification capability of a model is to calculate the receiver operating characteristic (ROC) curve and the area under curve (AUC).^36^Table 1 contains the experimental data that classifies triclosan derivatives as active (Y) or not active (N). These classification data are submitted along with the calculated pEC_50_ data to the *roc.curve* function of the Scikit-learn and Matplotlib libraries.^37^ In addition to the AUC, this analysis can determine the threshold based on theoretical values.

### 2.5 Web Software for pEC_50_ and EC_50_ Calculation

A software was built to allow the calculation of pEC_50_ and EC_50_ values using NextJS,^38^ NestJS,^39^ and javascript/typescript.^40^ It was hosted at https://www.qsar.labioquim.fiocruz.br/ and named PlasmoQSAR.

## 3 Results and Discussion

After several rounds varying the arrangements of all descriptors in groups of three and the degrees of the model from 1 to 8 in PR, the optimal combination of descriptors D143, D312, and D470 was obtained at degree 4, with *R*^2^ greater than 80% for internal (training) and external (testing) validation (Table 2). This model was selected for the construction of the analytical equation for the pEC_50_ calculation model. Where, D143 descriptor, is the sum of atomic van der Waals carbon-scale,^30^ D312 descriptor, is the sum eigenvalue weighted by electronegativity Pauling-scale distance matrix.^41^ And the D470 descriptor, is the Geary topological structure autocorrelation length-8 weighted by atomic Sanderson electronegativities. The Geary index is an important indicator of spatial autocorrelation being weighted by atomic electronegativities of Sanderson.^42^ In Table 2, we observe the description that encompasses the statistical parameter results, highlighting the best *R*^2^ for a specific training and testing group while considering the polynomial degree of 4 tested with the D143, D312, and D470 descriptors.

**Table 2:** Values of *R*^2^, *R*_pred_^2^, *Q*^2^, and Y-Randomization (*R*^2^) of the Models of Degree 1 to 8 Applied to the Descriptors D143, D312, and D470

degrees	*R*^2^(training) (%)	*R*_pred_^2^(test) (%)	*Q*^2^(LOO) (%)	*Q*^2^(LNO) (%)	Y-randomization (*R*^2^) (%)
1	37.23	52.11	37.27		–38.49
2	55.50	58.88	55.59		–55.62
3	70.22	70.32	70.42		–81.09
4	81.15	80.05	81.28	81.93	–94.10
5	91.52	–487.91			
6	99.67	–739965.34			
7	99.66	–25091.23			
8	99.63	–26369.02			

The average obtained from the *Q*^2^ for LOO and LNO in degree 4 of 81.28% and 81.93% respectively, shows a highly robust model. In the Y-randomization, negative *R*^2^ values were obtained in all randomizations performed, showing that only the original model presents significant statistical parameters and not by chance. The other degrees were not calculated as they did not pass either internal or external validation. The degree 3 model can also be considered a good model with *R*^2^ greater than 70% in all metrics. Nevertheless, our preference is always for a model that approaches the ideal. In the case of other degrees, both in testing and training sets, it was possible to achieve satisfactory model results, but with low significance. This may hinder the development of a calculation protocol and the testing of new drugs.

The error metrics obtained in Table 3, show relatively low values for the data sets, ensuring reliable predictive quality. The MAE metric based on the study by Roy et al.^43^ sets a threshold for poor predictions: MAE > 0.15 × training set range or MAE + 3 × σ ≤ 0.25 × training set range. In this article, the MAE values of both the training and test sets fall within the limits established by Roy et al.,^43^ demonstrating adequate model predictions. The RMSE displays slightly higher values than the MAE, as reported by Veerasamy et al.,^32^ who explain that this difference arises due to the variability in the magnitude of errors.

**Table 3:** MAE and RMSE Error Statistics for Training and Test Sets

set	range	MAE	RMSE
training	3.32	0.28	0.39
test	3.43	0.32	0.42

Equation 5 is the analytical solution of the model with the independent variables *A*, *B*, and *C* corresponding to the descriptors D143, D312, and D470 respectively, that predicts the pEC_50_ of all molecules (Tables 4 and S1). Details of each member of the equation calculated separately can be seen in the spreadsheet deposited in Supporting Information (Table S2).
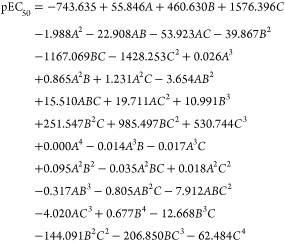
5

**Table 4:** All Molecules Submitted to the Equation, the Descriptors D143, D312, and D470 Calculated by the Mold2 Software, Observed and Calculated pEC_50_ Values as Well as the Module of the Differences

*N*	molecules (CID)	D143	D312	D470	set	pEC_50_ experimental	pEC_50_ calculated	Δ
**1**	5564	19.164	1.097	0.856	test	5.55	4.10	–1.45
**2**	44405339	22.359	1.485	0.775	test	4.00	4.22	–0.22
**3**	71552938	34.452	2.626	0.564	training	7.09	6.86	0.23
**4**	92602	19.559	0.999	0.859	training	4.28	4.40	–0.12
**5**	71364024	23.879	1.528	0.764	training	4.42	4.31	0.11
**6**	71552773	29.583	1.528	0.724	training	5.33	5.27	0.06
**7**	71552774	22.977	1.258	0.925	training	4.48	4.98	–0.50
**8**	71552860	36.343	2.304	0.566	training	6.21	6.30	–0.09
**9**	71552937	35.633	3.543	0.569	test	6.89	6.67	0.22
**10**	71552863	34.966	3.575	0.570	test	7.43	7.34	0.09
**11**	71552864	34.522	3.741	0.585	training	6.89	6.88	0.01
**12**	71552865	36.645	3.640	0.503	training	6.74	6.75	–0.01
**13**	71552935	36.063	4.060	0.569	training	7.14	7.15	–0.01
**14**	55224596	19.856	0.999	0.740	training	4.70	4.99	–0.29
**15**	63461798	20.879	1.355	0.739	training	3.82	4.15	–0.33
**16**	71552862	31.269	1.805	0.772	training	5.72	5.70	0.02
**17**	71718729	28.669	0.999	0.805	training	5.01	5.19	–0.18
**18**	71552776	22.384	1.419	0.865	training	4.74	4.40	0.34
**19**	71552778	36.343	2.304	0.610	training	5.92	5.94	–0.02
**20**	627458	20.868	1.097	0.612	training	4.00	4.83	–0.83
**21**	71552936	35.337	3.543	0.587	training	5.82	5.82	0.00
**22**	71552939	33.118	2.690	0.563	training	6.70	6.84	–0.14
**23**	71552690	21.274	1.258	0.721	training	4.14	4.26	–0.12
**24**	71552691	24.669	0.999	0.586	training	5.14	5.33	–0.19
**25**	71552861	30.530	1.612	0.784	test	5.70	5.75	–0.05
**26**	55193760	21.263	0.999	0.775	test	5.64	5.15	0.49
**27**	71552777	29.791	1.419	0.809	test	6.14	5.71	0.43
**28**	44405271	30.360	1.323	0.768	training	5.12	5.38	–0.26
**29**	44405274	24.656	1.323	0.441	test	4.16	3.95	0.21
**30**	44405275	27.360	1.323	0.363	training	4.68	4.54	0.14
**31**	44405276	29.063	1.323	0.420	training	4.93	4.97	–0.04
**32**	44405287	29.558	1.594	0.691	training	4.26	5.15	–0.89
**33**	44405289	34.262	1.594	0.831	training	4.46	4.66	–0.20
**34**	44405291	28.075	1.582	0.334	training	4.20	4.32	–0.12
**35**	11674015	22.953	1.323	0.646	test	4.28	4.08	0.20
**36**	44405293	28.656	1.323	0.683	training	4.24	4.63	–0.39
**37**	44405298	21.964	1.582	0.686	test	4.51	4.14	0.37
**38**	6914565	22.953	1.323	0.561	test	4.11	4.00	0.11
**39**	21272512	19.140	1.162	0.859	training	4.19	4.07	0.12
**40**	6852143	20.261	1.582	0.251	training	5.68	5.69	–0.01
**41**	6852148	19.535	1.065	1.197	training	3.92	4.02	–0.10
**42**	11659169	19.831	1.065	0.596	training	5.41	4.75	0.66
**43**	44405311	21.535	1.065	1.105	test	4.64	5.17	–0.53
**44**	11660481	28.645	1.065	0.748	training	4.45	4.93	–0.48
**45**	44405314	30.051	1.226	0.713	training	4.57	4.93	–0.36
**46**	44405327	20.855	1.421	0.653	training	3.96	4.06	–0.10
**47**	44405330	22.457	1.548	0.520	training	3.92	3.76	0.16
**48**	44405331	21.250	1.323	0.763	training	4.02	4.21	–0.19
**49**	44405336	34.262	1.594	0.804	test	4.63	5.01	–0.38
**50**	44405338	24.739	2.672	0.741	training	3.92	3.92	0.00
**51**	6914566	29.184	1.743	0.519	training	4.18	3.95	0.23
**52**	44405380	29.360	1.323	0.771	training	5.35	5.29	0.06
**53**	44410066	32.791	1.258	0.842	training	6.60	5.80	0.80
**54**	44410081	33.016	0.872	0.880	training	6.49	6.35	0.14
**55**	44410086	32.609	0.872	0.842	test	6.52	6.04	0.48
**56**	11948630	36.198	1.258	0.734	test	6.85	6.70	0.15
**57**	44410094	34.494	1.258	0.712	training	6.39	6.18	0.21
**58**	44410097	34.506	1.516	0.744	test	5.46	5.58	–0.12
**59**	44410098	22.942	1.065	1.057	training	6.24	5.79	0.45
**60**	44410128	32.609	0.872	0.751	training	6.35	6.23	0.12
**61**	44410129	21.238	1.065	0.798	training	5.57	4.80	0.77
**62**	44410130	30.348	1.065	0.847	test	6.11	5.63	0.48
**63**	44410133	30.360	1.323	0.759	test	5.07	5.32	0.25
**64**	44410134	24.645	1.065	1.075	training	6.74	6.78	–0.04
**65**	44410138	27.348	1.065	0.987	training	6.30	6.87	–0.57
**66**	44410170	31.262	1.594	0.755	test	4.96	5.76	–0.80
**67**	44410210	37.755	1.065	0.910	training	6.64	6.57	0.07
**68**	44410211	33.348	1.065	0.756	training	5.59	5.94	–0.35
**69**	44410215	44.975	1.226	0.831	training	5.68	5.66	0.02
**70**	44410233	29.052	1.065	0.950	test	6.14	6.67	–0.53
**71**	44410234	35.865	1.226	0.829	training	4.85	5.66	–0.81
**72**	44410238	37.569	1.226	0.823	test	6.20	5.69	0.51
**73**	44410251	18.796	0.872	0.920	test	4.08	3.95	0.13
**74**	44410252	20.499	0.872	0.846	test	5.01	5.26	–0.25
**75**	44410256	31.088	1.258	0.831	training	6.49	5.78	0.71
**76**	44410284	29.609	0.872	0.908	training	6.31	5.78	0.53
**77**	44410286	30.348	1.065	0.886	training	6.48	6.00	0.48
**78**	44410287	31.313	0.872	0.872	training	6.11	5.74	0.37
**79**	44410291	33.348	1.065	0.711	test	6.68	6.15	0.53
**80**	11495355	33.767	1.323	0.761	training	6.74	5.67	1.07
**81**	44410295	32.052	1.065	0.816	training	4.72	5.61	–0.89
**82**	23656593	20.855	1.421	0.652	test	4.00	4.06	–0.06
**83**	25023956	22.457	1.548	0.519	test	4.00	3.76	0.24
**84**	25023957	21.250	1.323	0.762	training	4.40	4.21	0.19
**85**	25023955	19.831	1.065	0.594	test	4.72	4.75	–0.03
**86**	22947105	20.128	0.904	1.376	training	5.00	4.96	0.04
**87**	25023954	25.832	0.904	0.742	test	5.64	5.50	0.14
**88**	25023973	27.609	1.033	0.649	training	5.13	4.86	0.27
**89**	16220130	26.942	1.065	0.787	training	5.12	5.10	0.02
**90**	25023972	26.942	1.065	0.836	test	5.16	5.38	–0.22
**91**	25023971	25.905	1.033	0.703	training	4.96	5.01	–0.05
**92**	16220129	25.905	1.033	0.523	test	5.09	4.95	0.14
**93**	25023970	25.238	1.065	0.745	training	4.64	5.01	–0.37
**94**	16220126	30.942	0.904	0.896	training	5.19	5.89	–0.70
**95**	25023969	27.535	0.904	0.780	training	4.96	5.23	0.27
**96**	16220128	29.239	0.904	0.840	training	5.25	5.33	–0.08
**97**	25023967	27.535	0.904	0.712	test	5.54	5.26	0.28
**98**	25023966	27.535	0.904	0.540	training	5.46	5.54	–0.08
**99**	25023968	26.127	1.263	0.698	training	5.68	4.47	1.21
**100**	25023964	29.646	0.904	0.679	training	5.47	5.31	0.16
**101**	15942656	27.535	0.904	0.579	training	5.80	5.52	0.28
**102**	25023965	28.202	0.872	0.512	training	5.59	5.74	–0.15
**103**	25023962	26.942	0.904	0.697	training	5.70	5.38	0.32
**104**	25023963	26.942	0.904	0.669	test	5.13	5.43	–0.30
**105**	25023961	25.239	0.904	0.816	test	5.70	5.50	0.20
**106**	25023959	23.535	0.904	0.972	training	5.60	5.52	0.08
**107**	25023960	25.239	0.904	0.805	training	5.46	5.50	–0.04
**108**	25023958	21.832	0.904	1.162	training	5.34	5.46	–0.12

**Figure 2:**
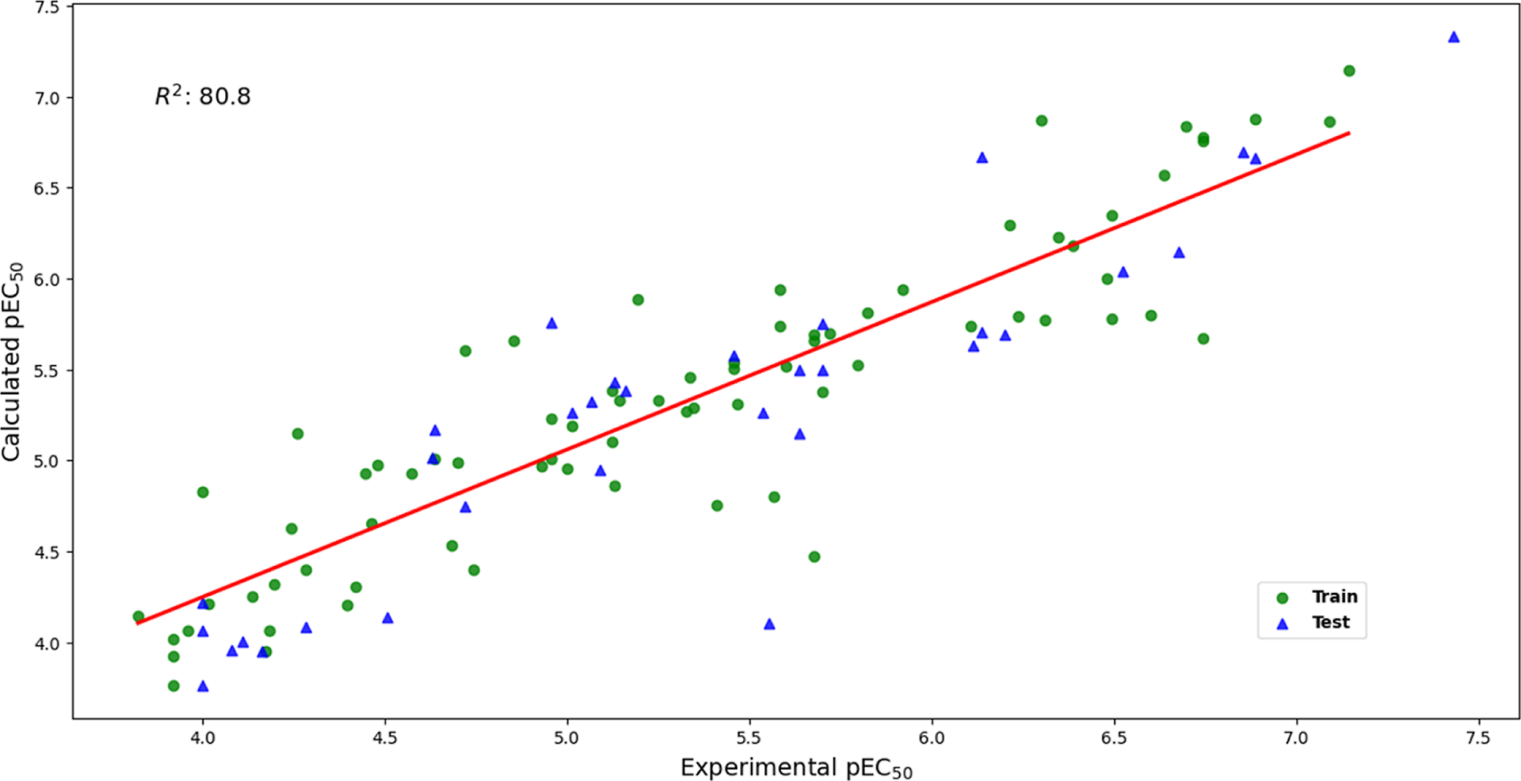
Correlation graph between the observed data versus the calculated (predicted) values.

**Figure 3:**
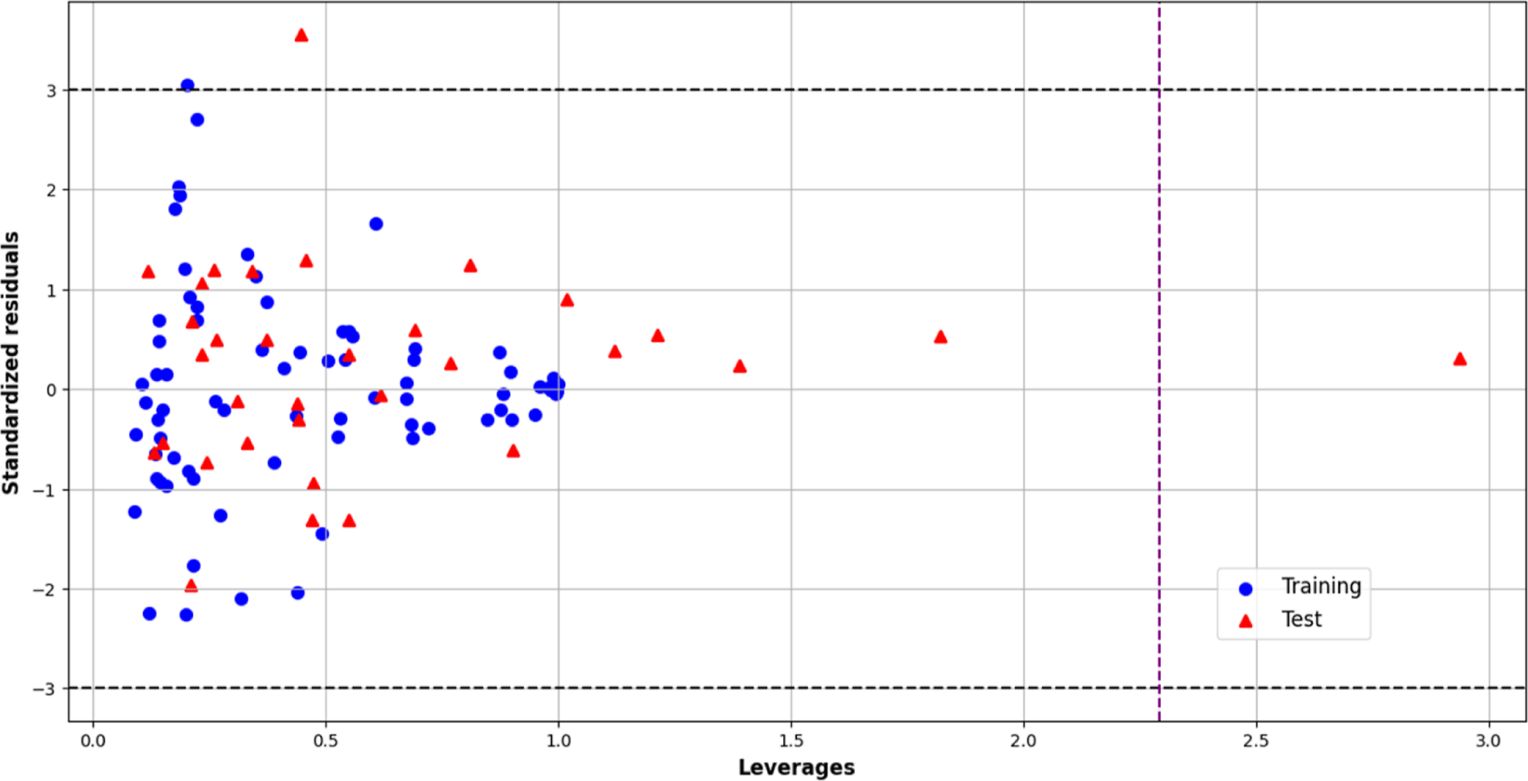
Williams plot, with standardized residual values and leverage values to identify outliers and influential points, respectively.

The regression result in Figure 2 demonstrates a high coefficient of determination (*R*^2^ = 80.8), indicating a strong correlation between all pEC_50_ observed data and predicted values.^44^ The value of *R*^2^ on the line between the observed and predicted pEC_50_ values, also determine a good correlation, corroborating the average *R*^2^ of the internal and external validations of the model. In addition, the high correlation of the plotted data indicates a model with favorable performance to predict antimalarial activity of any compound of interest derived from triclosan. Outlier identification and leverage values of training and test compounds were plotted on the Williams plot (Figure 3) to assess the applicability domain of the model. Leverage and residual outliers are useful for identifying influential and unusual points, respectively,^45^ and for assessing the model’s reliability with respect to a specific prediction.^46^

Thus, Figure 3 shows that only two compounds (one from training and one from testing) are outliers. Regarding leverage, only one compound from the test group exceeded the defined threshold.

**Figure 4:**
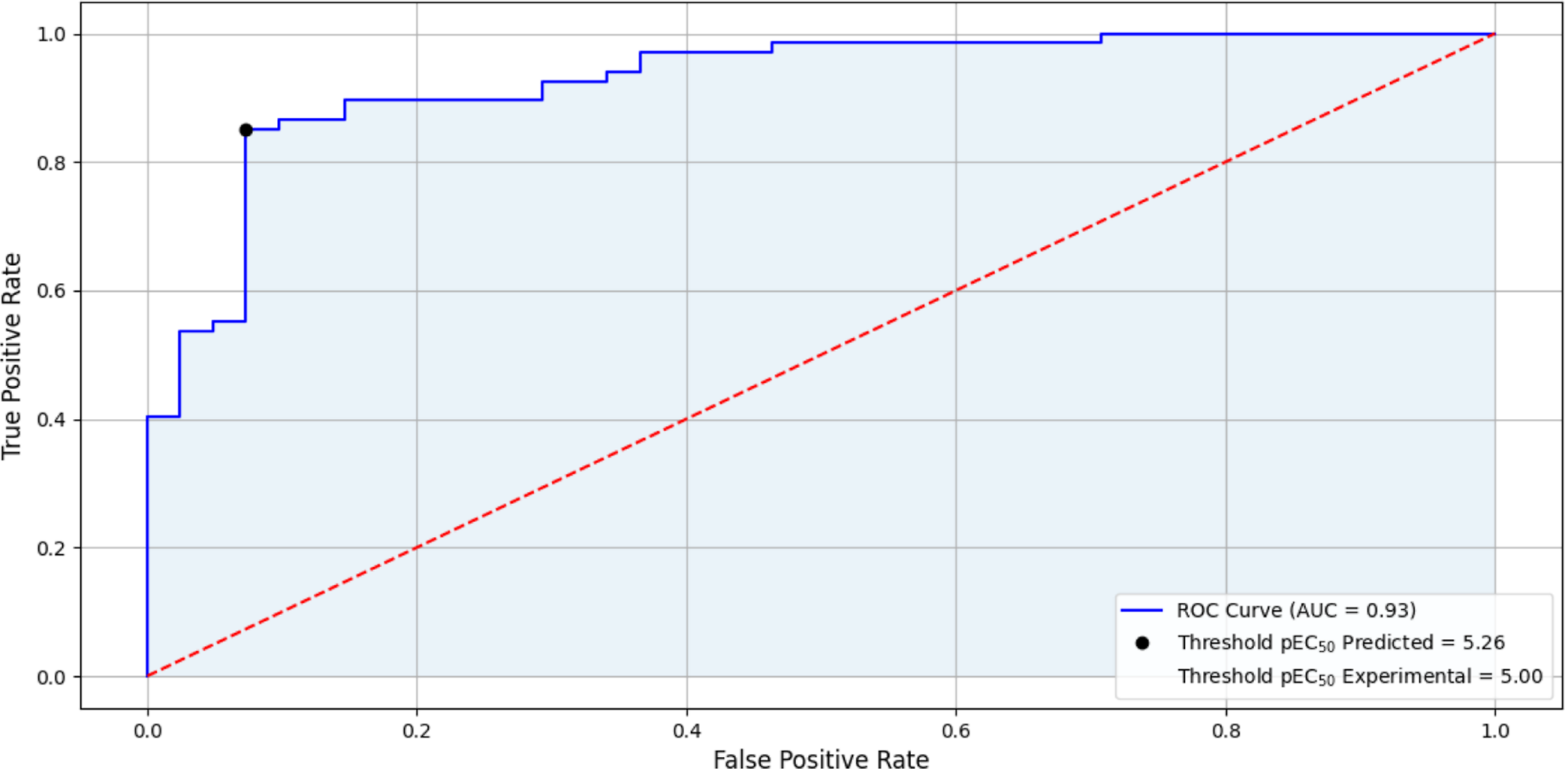
ROC curve of the pEC_50_ values calculated by applying eq 3 of the model.

The ROC curve plotted in Figure 4, has a common evaluation in binary evaluation tests that allows, in this case, to evaluate the model’s ability to predict between active and inactive compounds. In addition to the rate of true positives and false positives, the ROC curve also allows predicting the limit of the pEC_50_ calculated by the model. In this case, the limit resulted in pEC_50_ = 5.26, which converted into EC_50_ is equal to 5.49 μM. The limit value for the experimental data used in the four studies^26−29^ and indicated in the PubChem was EC_50_ = 5.00 μM corresponding to a pEC_50_ = 10.00.

**Figure 5:**
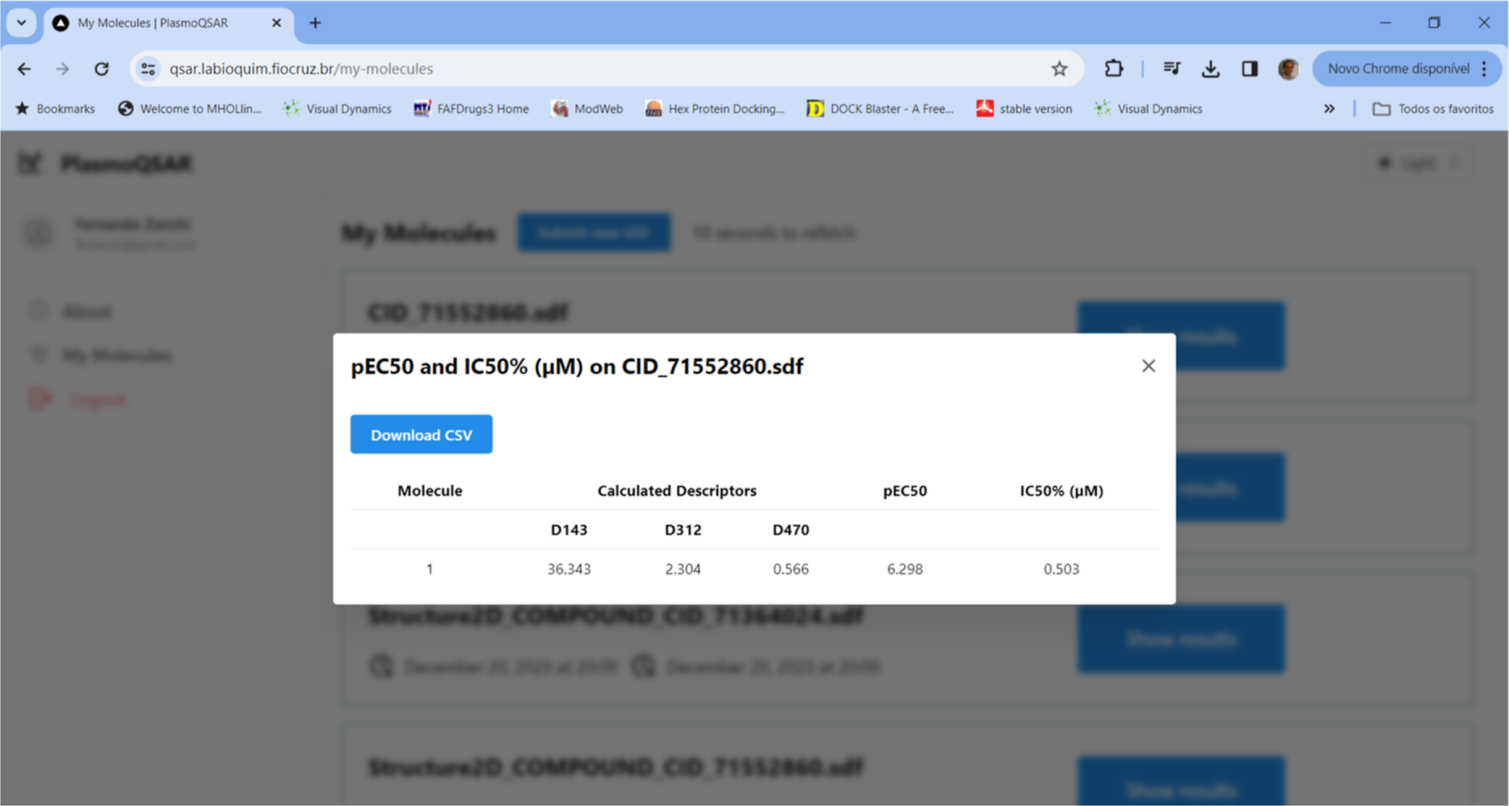
QSAR model calculation result for compound CID 71552860 on PlasmoQSAR.

To calculate the values of any molecules, simply access the PlasmoQSAR software and upload an SDF file containing the molecules. The software will calculate the D143, D312, and D470 descriptors for the molecules and will submit the model equation that will calculate the pEC_50_ and EC_50_ (Figure 5).

## 4 Conclusion

In summary, the application of ML in Python for the QSAR model targeting triclosan derivatives against the malaria-causing parasite *P. falciparum* 3D7, utilizing 777 descriptors grouped in sets of three, resulted in a fourth-degree analytical PR model with an *R*^2^ exceeding 80%, showcasing promising outcomes. This approach underscores the use of free libraries to evaluate QSAR models with higher degrees, streamlining the proposal of new drug tests for more effective malaria treatment.

Beyond the successful model, we introduced the PlasmoQSAR software, a free web service enabling researchers to submit molecules for evaluating their compatibility with antimalarial inhibitors. This tool enhances the understanding of structural factors influencing triclosan activity. The integration of this tool with advanced techniques such as ML and QSAR emerges as an effective approach for discovering and evaluating potential candidates for antimalarial medications, demonstrating the practical applicability of these methodologies in new drug research. Overall, this work significantly contributes to comprehending and practically applying QSAR in antimalarial studies, propelling advancements in the development of novel therapies against malaria.

## Data Availability

The source code of the python program, two files with calculated descriptors to train and test the model, two SDF files of the training and testing molecules are on Zenodo^47^ repository. CID is the registered number in the database of PubChem. The Mold2 software used to calculate the descriptors is publicly available at https://www.fda.gov/science-research/bioinformatics-tools/mold2. PlasmoQSAR Web is an open-source software project, publicly available at https://www.qsar.labioquim.fiocruz.br/ and can be downloaded from the GitHub repository at https://github.com/LABIOQUIM/plasmoqsar.

## Supporting Information Available

Additional data on all triclosan-derived molecules, including structures, are in Table S1. The calculated descriptors and the complete equation with all coefficients are in an Excel spreadsheet in Table S2 (PDF)

## Notes

All authors read and approved for publication.
