# Supplementary material for: Application of Machine Learning in the Development of Fourth Degree Quantitative Structure–Activity Relationship Model for Triclosan Analogs Tested against Plasmodium falciparum 3D7
Source: ACS Omega. 2024 Oct 25;9(44):44436–47. doi: 10.1021/acsomega.4c05768 (PMC11541538; doi:10.1021/acsomega.4c05768)
Supplement: Supplementary file 1 — ao4c05768_si_001.pdf [file ao4c05768_si_001.pdf]

Supporting Information

Application of machine learning in the development of 4th degree QSAR model for triclosan analogs tested against *P. falciparum* 3D7

Railton Marques de Souza Guimarães[1,2], Ivo Henrique Provensi Vieira[1,7], Fabrício Berton Zanchi[3], Rafael Andrade Caceres[4], Fernando Berton Zanchi[1,5,6,7]*

[1]Laboratório de Bioinformática e Química Medicinal, Fundação Oswaldo Cruz Rondônia, Porto Velho-RO, Brazil;

[2]Centro Universitário Afya, Faculdade de Biomedicina, Porto Velho-RO, Brazil;

[3]Centro de Formação em Ciências Ambientais-CFCAm da Universidade Federal do Sul da Bahia-UFSB, Porto Seguro-BA, Brasil

[4]Laboratório de Bioinformática Estrutural, Modelagem Molecular e Simulação de Biossistemas - Programa de Pós-Graduação em Biociências - Universidade Federal de Ciências da Saúde de Porto Alegre – UFCSPA, Porto Alegre RS, Brazil

[5]Programa de Pós-Graduação em Biologia Experimental, FIOCRUZ Rondônia / Universidade Federal de Rondônia (UNIR), Porto Velho-RO, Brasil

[6]Programa de Pós-Graduação Rede BIONORTE, FIOCRUZ Rondônia, Porto Velho-RO, Brasil

[7]Instituto Nacional de Epidemiologia na Amazônia Ocidental - EPIAMO, Porto Velho-RO, Brasil;

***Corresponding author: fernando.zanchi@fiocruz.br**

Table S1. All molecules submitted to the equation, the descriptors D143, D312, and D470 calculated by the Mold2 software, observed and calculated pEC50 values as well as the module of the differences. CID and structure are retrieved from Pubchem data.

N	CID	Structure	D143	D312	D470	pEC50 experimental	pEC50 calculated
**1**	5564	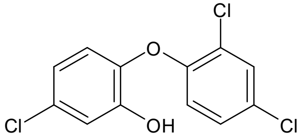	19.164	1.097	0.856	5.55	4.10
**2**	44405339	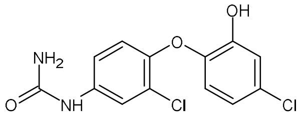	22.359	1.485	0.775	4.00	4.22
**3**	71552938	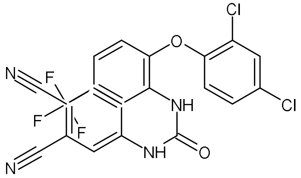	34.452	2.626	0.564	7.09	6.86
**4**	92602	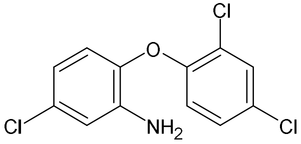	19.559	0.999	0.859	4.28	4.40
**5**	71364024	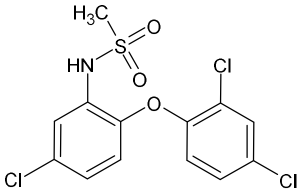	23.879	1.528	0.764	4.42	4.31
**6**	71552773	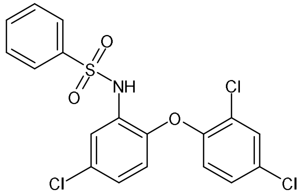	29.583	1.528	0.724	5.33	5.27

**7**	71552774	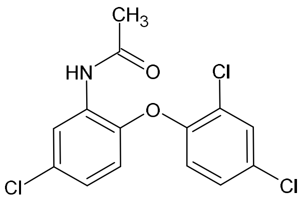	22.977	1.258	0.925	4.48	4.98
**8**	71552860	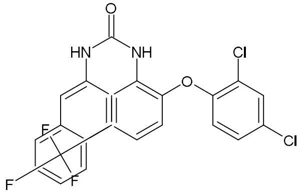	36.343	2.304	0.566	6.21	6.30
**9**	71552937	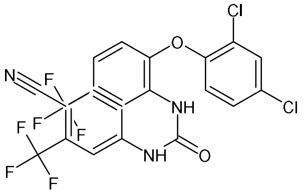	35.633	3.543	0.569	6.89	6.67
**10**	71552863	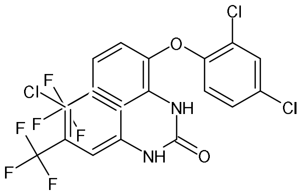	34.966	3.575	0.57	7.43	7.34
**11**	71552864	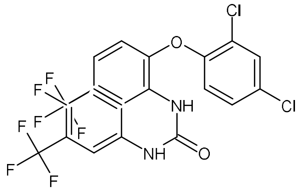	34.522	3.741	0.585	6.89	6.88
**12**	71552865	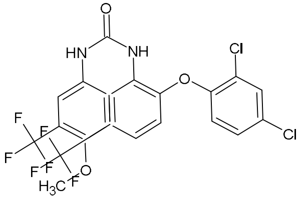	36.645	3.64	0.503	6.74	6.75

**13**	71552935	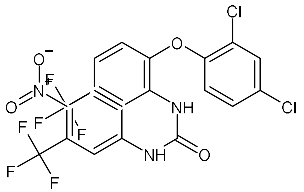	36.063	4.06	0.569	7.14	7.15
**14**	55224596	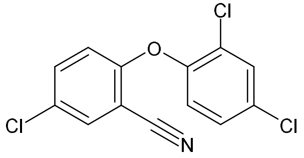	19.856	0.999	0.74	4.70	4.99
**15**	63461798	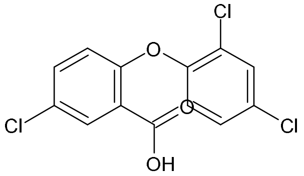	20.879	1.355	0.739	3.82	4.15
**16**	71552862	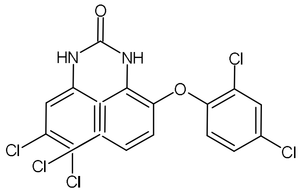	31.269	1.805	0.772	5.72	5.70
**17**	71718729	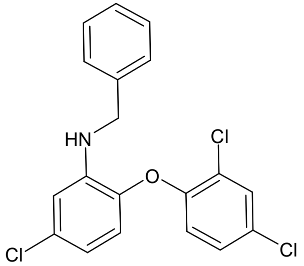	28.669	0.999	0.805	5.01	5.19
**18**	71552776	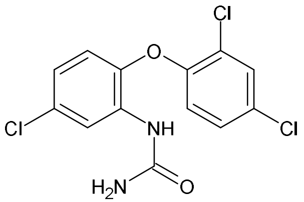	22.384	1.419	0.865	4.74	4.40

**19**	71552778	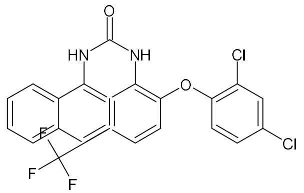	36.343	2.304	0.61	5.92	5.94
**20**	627458	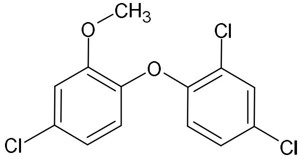	20.868	1.097	0.612	4.00	4.83
**21**	71552936	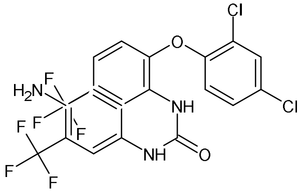	35.337	3.543	0.587	5.82	5.82
**22**	71552939	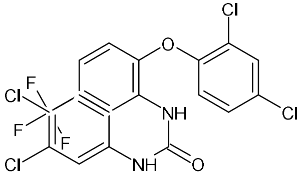	33.118	2.69	0.563	6.70	6.84
**23**	71552690	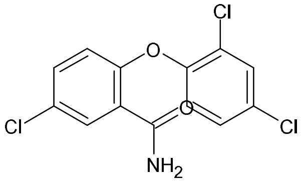	21.274	1.258	0.721	4.14	4.26
**24**	71552691	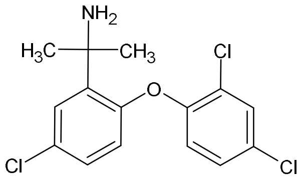	24.669	0.999	0.586	5.14	5.33

**25**	71552861	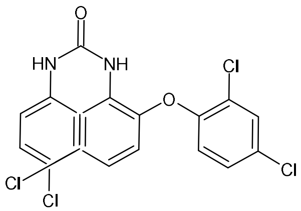	30.53	1.612	0.784	5.70	5.75
**26**	55193760	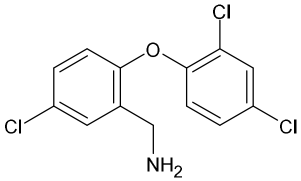	21.263	0.999	0.775	5.64	5.15
**27**	71552777	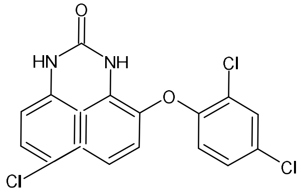	29.791	1.419	0.809	6.14	5.71
**28**	44405271	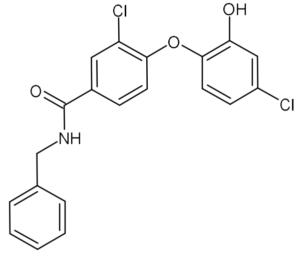	30.36	1.323	0.768	5.12	5.38
**29**	44405274	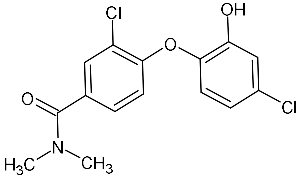	24.656	1.323	0.441	4.16	3.95
**30**	44405275	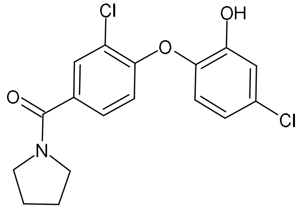	27.36	1.323	0.363	4.68	4.54

**31**	44405276	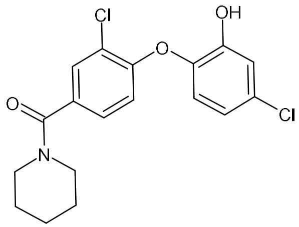	29.063	1.323	0.42	4.93	4.97
**32**	44405287	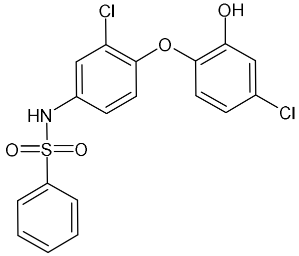	29.558	1.594	0.691	4.26	5.15
**33**	44405289	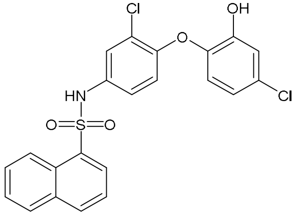	34.262	1.594	0.831	4.46	4.66
**34**	44405291	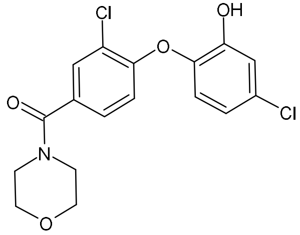	28.075	1.582	0.334	4.20	4.32
**35**	11674015	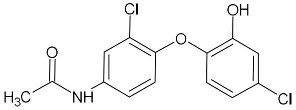	22.953	1.323	0.646	4.28	4.08
**36**	44405293	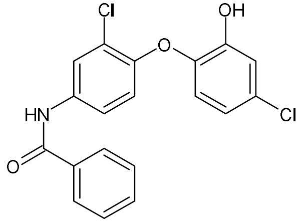	28.656	1.323	0.683	4.24	4.63

**37**	44405298	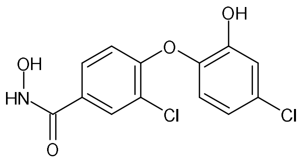	21.964	1.582	0.686	4.51	4.14
**38**	6914565	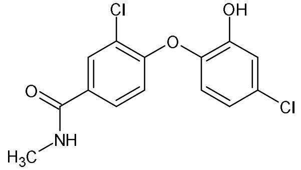	22.953	1.323	0.561	4.11	4.00
**39**	21272512	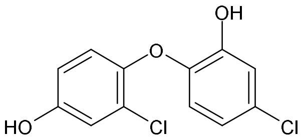	19.14	1.162	0.859	4.19	4.07
**40**	6852143	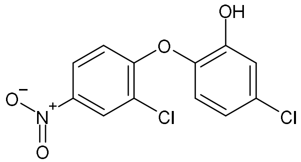	20.261	1.582	0.251	5.68	5.69
**41**	6852148	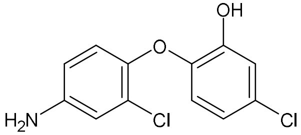	19.535	1.065	1.197	3.92	4.02
**42**	11659169	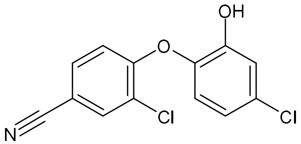	19.831	1.065	0.596	5.41	4.75
**43**	44405311	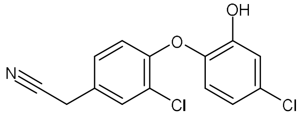	21.535	1.065	1.105	4.64	5.17
**44**	11660481	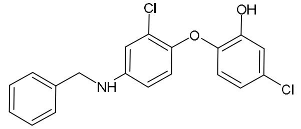	28.645	1.065	0.748	4.45	4.93

**45**	44405314	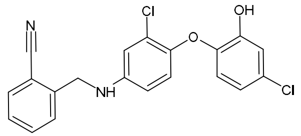	30.051	1.226	0.713	4.57	4.93
**46**	44405327	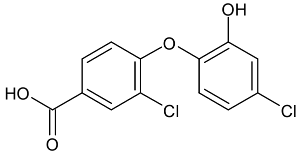	20.855	1.421	0.653	3.96	4.06
**47**	44405330	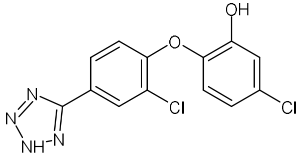	22.457	1.548	0.52	3.92	3.76
**48**	44405331	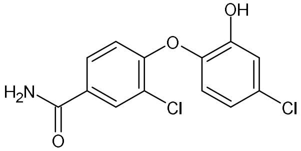	21.25	1.323	0.763	4.02	4.21
**49**	44405336	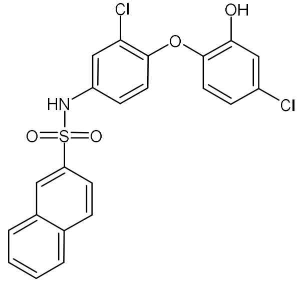	34.262	1.594	0.804	4.63	5.01
**50**	44405338	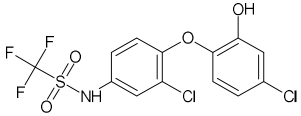	24.739	2.672	0.741	3.92	3.92
**51**	6914566	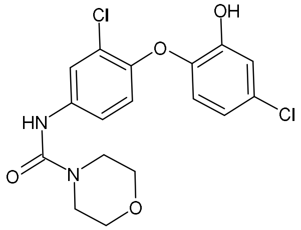	29.184	1.743	0.519	4.18	3.95

**52**	44405380	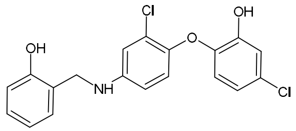	29.36	1.323	0.771	5.35	5.29
**53**	44410066	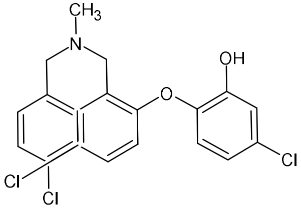	32.791	1.258	0.842	6.60	5.80
**54**	44410081	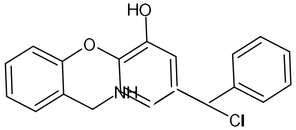	33.016	0.872	0.88	6.49	6.35
**55**	44410086	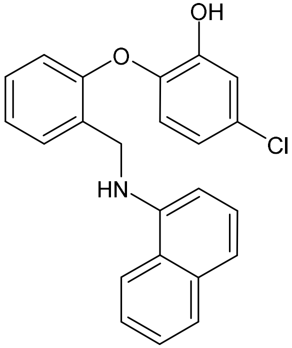	32.609	0.872	0.842	6.52	6.04
**56**	11948630	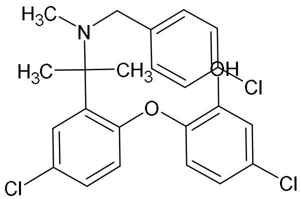	36.198	1.258	0.734	6.85	6.70

**57**	44410094	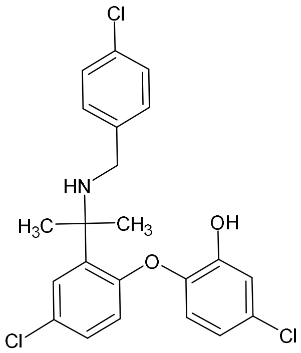	34.494	1.258	0.712	6.39	6.18
**58**	44410097	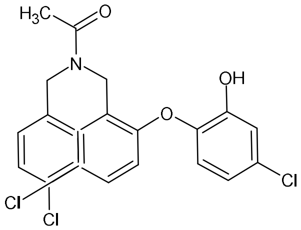	34.506	1.516	0.744	5.46	5.58
**59**	44410098	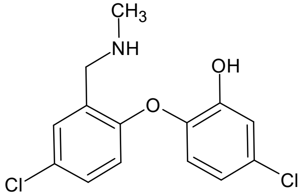	22.942	1.065	1.057	6.24	5.79
**60**	44410128	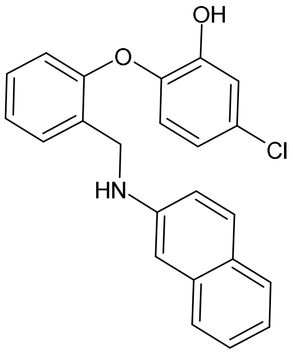	32.609	0.872	0.751	6.35	6.23

**61**	44410129	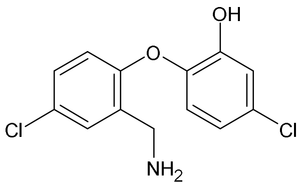	21.238	1.065	0.798	5.57	4.80
**62**	44410130	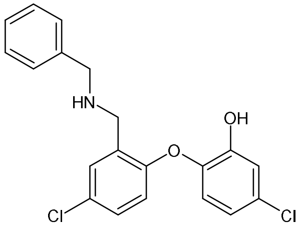	30.348	1.065	0.847	6.11	5.63
**63**	44410133	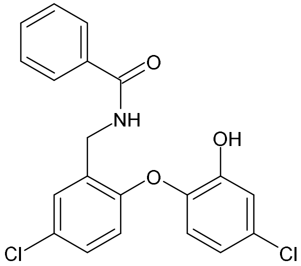	30.36	1.323	0.759	5.07	5.32
**64**	44410134	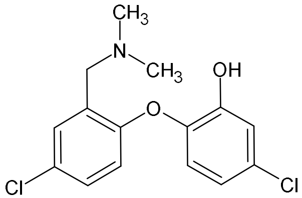	24.645	1.065	1.075	6.74	6.78
**65**	44410138	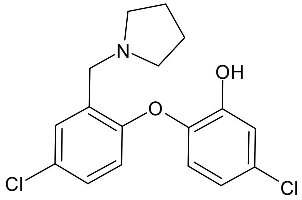	27.348	1.065	0.987	6.30	6.87

**66**	44410170	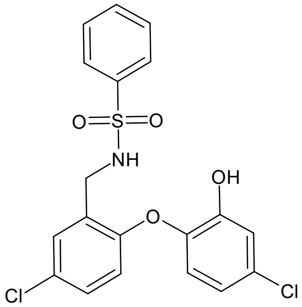	31.262	1.594	0.755	4.96	5.76
**67**	44410210	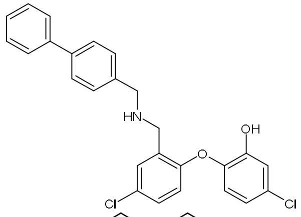	37.755	1.065	0.91	6.64	6.57
**68**	44410211	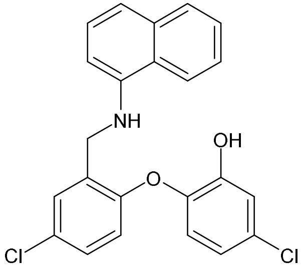	33.348	1.065	0.756	5.59	5.94
**69**	44410215	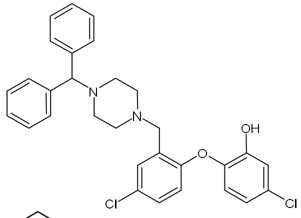	44.975	1.226	0.831	5.68	5.66
**70**	44410233	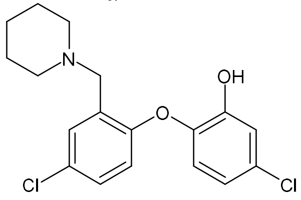	29.052	1.065	0.95	6.14	6.67

**71**	44410234	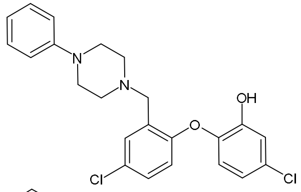	35.865	1.226	0.829	4.85	5.66
**72**	44410238	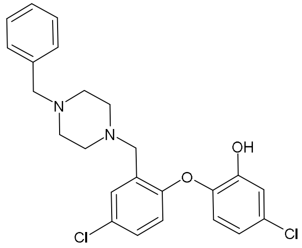	37.569	1.226	0.823	6.20	5.69
**73**	44410251	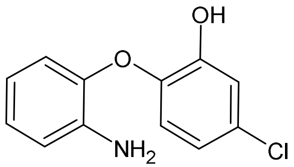	18.796	0.872	0.92	4.08	3.95
**74**	44410252	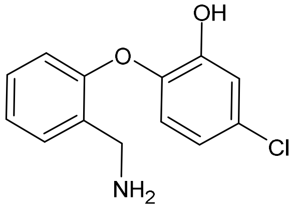	20.499	0.872	0.846	5.01	5.26
**75**	44410256	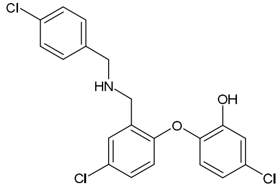	31.088	1.258	0.831	6.49	5.78
**76**	44410284	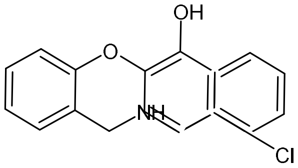	29.609	0.872	0.908	6.31	5.78

**77**	44410286	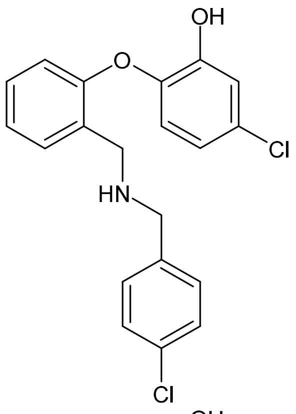	30.348	1.065	0.886	6.48	6.00
**78**	44410287	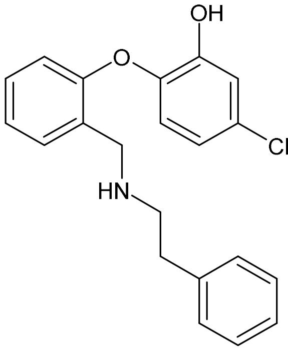	31.313	0.872	0.872	6.11	5.74
**79**	44410291	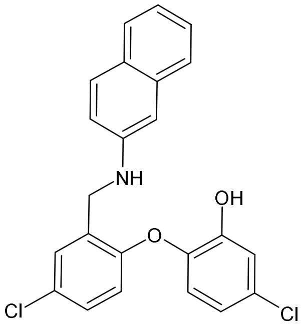	33.348	1.065	0.711	6.68	6.15

**80**	11495355	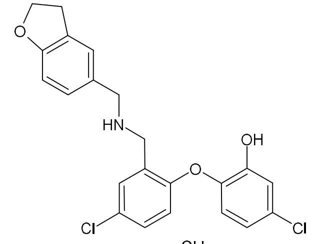	33.767	1.323	0.761	6.74	5.67
**81**	44410295	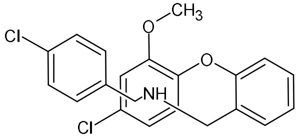	32.052	1.065	0.816	4.72	5.61
**82**	23656593	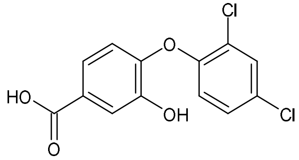	20.855	1.421	0.652	4.00	4.06
**83**	25023956	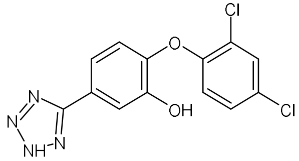	22.457	1.548	0.519	4.00	3.76
**84**	25023957	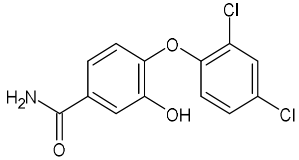	21.25	1.323	0.762	4.40	4.21
**85**	25023955	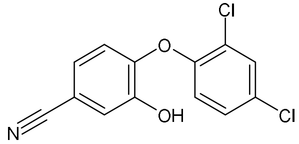	19.831	1.065	0.594	4.72	4.75
**86**	22947105	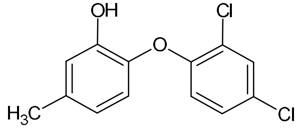	20.128	0.904	1.376	5.00	4.96
**87**	25023954	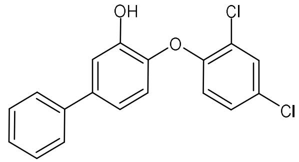	25.832	0.904	0.742	5.64	5.50

**88**	25023973	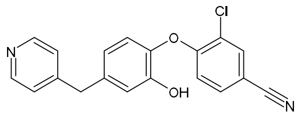	27.609	1.033	0.649	5.13	4.86
**89**	16220130	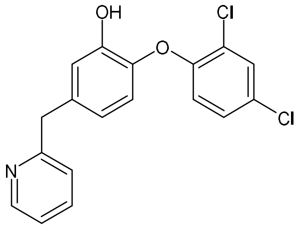	26.942	1.065	0.787	5.12	5.10
**90**	25023972	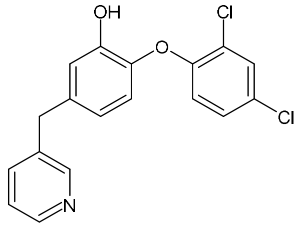	26.942	1.065	0.836	5.16	5.38
**91**	25023971	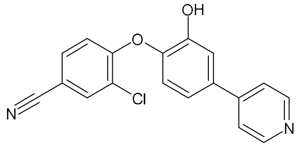	25.905	1.033	0.703	4.96	5.01
**92**	16220129	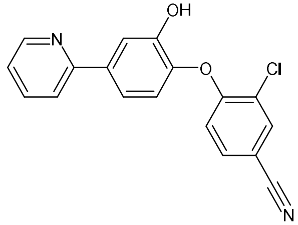	25.905	1.033	0.523	5.09	4.95
**93**	25023970	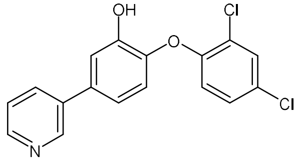	25.238	1.065	0.745	4.64	5.01

**94**	16220126	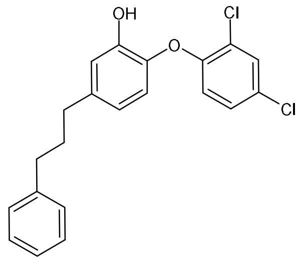	30.942	0.904	0.896	5.19	5.89
**95**	25023969	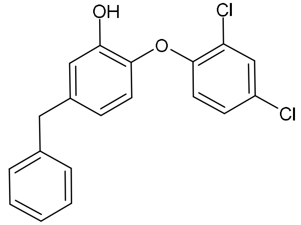	27.535	0.904	0.78	4.96	5.23
**96**	16220128	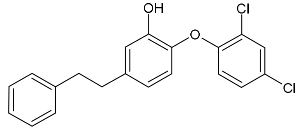	29.239	0.904	0.84	5.25	5.33
**97**	25023967	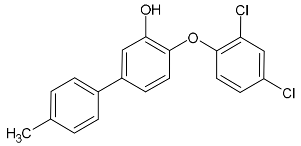	27.535	0.904	0.712	5.54	5.26
**98**	25023966	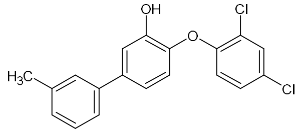	27.535	0.904	0.54	5.46	5.54
**99**	25023968	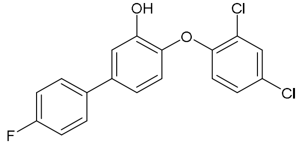	26.127	1.263	0.698	5.68	4.47
**100**	25023964	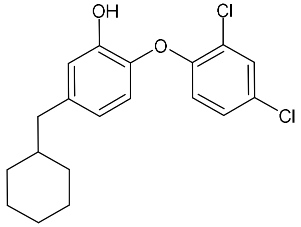	29.646	0.904	0.679	5.47	5.31

**101**	15942656	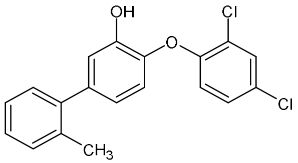	27.535	0.904	0.579	5.80	5.52
**102**	25023965	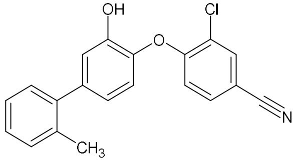	28.202	0.872	0.512	5.59	5.74
**103**	25023962	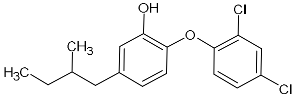	26.942	0.904	0.697	5.70	5.38
**104**	25023963	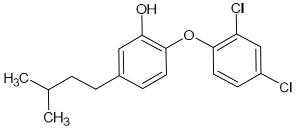	26.942	0.904	0.669	5.13	5.43
**105**	25023961	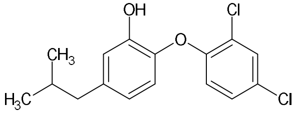	25.239	0.904	0.816	5.70	5.50
**106**	25023959	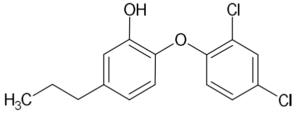	23.535	0.904	0.972	5.60	5.52
**107**	25023960	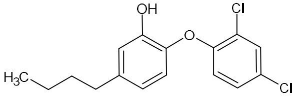	25.239	0.904	0.805	5.46	5.50
**108**	25023958	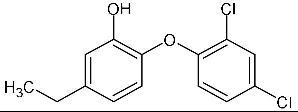	21.832	0.904	1.162	5.34	5.46

Table S2. Table containing each member of the equation separated in each column with multiplied coefficients and descriptors. Column AQ contains the Intercept value of the equation and column AS contains the value of the final PEC50.

		coefficients of members of equation ------------->				55,846 x	460,630 x	1576,396 x	-1,988 x	-22,908 x	-53,923 x	-39,867 x	-1167,069 x	-1428,253 x	0,026 x	0,865 x	1,231 x	-3,654 x	15,510 x	19,711 x	10,991 x	251,547 x	985,497 x	530,744 x
N	CID	PEC50	D143	D312	D470	[D143]	[D312]	[D470]	[D143^{2}]	[D143 x D312]	[D143 x D470]	[D312^{2}]	[D312 x D470]	[D470^{2}]	[D143^{3}]	[D143^{2} x D312]	[D143^{2} x D470]	[D143 x D312^{2}]	[D143 x D312 x D470]	[D143 x D470^{2}]	[D312^{3}]	[D312^{2} x D470]	[D312 x D470^{2}]	[D470^{3}]
1	5564	5,55284	19,16	1,097	0,856	1070,241272	505,310966	1349,39475	-730,091955	-481,5866588	-884,5693005	-47,9763896	-1095,91508	-1046,5323	180,781908	348,399931	386,8527663	-84,27993169	279,1192596	276,7859251	14,5100208	259,1230394	792,153713	332,894556
2	44405339	4,00000	22,36	1,485	0,775	1248,670664	684,035355	1221,70669	-993,825167	-760,6072963	-934,3853367	-87,9158572	-1343,150465	-857,844385	287,113495	641,9930313	476,7668732	-180,1897326	399,1202676	264,7074055	35,9937847	429,9061115	878,9924887	247,053213
3	71552938	7,09151	34,45	2,626	0,564	1924,021723	1209,61404	889,087193	-2359,57679	-2072,482241	-1047,76884	-274,918364	-1728,503791	-454,321528	1050,36392	2695,396792	823,773086	-868,2177085	791,428188	216,0145262	199,036313	978,3349235	823,2054461	95,2188014
4	92602	4,28400	19,56	0,999	0,859	1092,300618	460,169239	1354,12393	-760,498798	-447,6038285	-905,9656812	-39,7873747	-1001,509706	-1053,88066	192,192492	330,4896704	404,3766545	-71,33495321	260,3325958	284,474462	10,9583361	215,6468653	726,4523301	336,406896
5	71364024	4,42022	23,88	1,528	0,764	1333,55726	703,84244	1204,36634	-1133,54172	-835,8361439	-983,7424904	-93,0809948	-1362,426943	-833,665492	349,739661	753,4505143	536,0746211	-203,7453011	432,3705432	274,7344768	39,2119297	448,7031065	878,9524411	236,682133
6	71552773	5,32790	29,58	1,528	0,724	1652,105382	703,84244	1141,31051	-1739,7613	-1035,493138	-1154,922163	-93,0809948	-1291,095689	-748,655881	665,002256	1156,396822	779,6911611	-252,4141397	507,6067447	305,6536993	39,2119297	425,2107973	789,3248793	201,419307
7	71552774	4,48149	22,98	1,258	0,925	1283,183767	579,472375	1458,16605	-1049,52282	-662,1488406	-1146,059045	-63,0921882	-1358,059771	-1222,04887	311,584935	574,3364744	600,9356915	-132,8860413	414,7046911	387,5137349	21,8821543	368,2324218	1060,765562	420,059293
8	71552860	6,21467	36,34	2,304	0,566	2029,627351	1061,29122	892,239984	-2625,71017	-1918,160336	-1109,198162	-211,630987	-1521,932589	-457,549379	1232,98786	2631,619715	919,935963	-705,0346818	735,0940882	229,4900956	134,429808	755,7886923	727,3955542	96,2353637
9	71552937	6,88606	35,63	3,543	0,569	1989,976375	1632,01163	896,969172	-2524,12009	-2892,046009	-1093,293057	-500,445297	-2352,772468	-462,41258	1162,12713	3890,22783	889,0304983	-1634,629943	1114,189488	227,3983091	488,834231	1796,691778	1130,449043	97,7737332
10	71552863	7,43180	34,97	3,575	0,57	1952,726796	1646,75178	898,545567	-2430,5085	-2863,542657	-1074,7136	-509,526059	-2378,19473	-464,03936	1098,0809	3779,784684	857,5636811	-1633,137695	1105,147142	223,926749	502,199518	1832,508329	1144,672008	98,2901427
11	71552864	6,88606	34,52	3,741	0,585	1927,930974	1723,21634	922,191503	-2369,17497	-2958,457358	-1088,989645	-557,94286	-2554,112867	-488,783842	1056,77936	3855,482294	857,9211614	-1765,615457	1171,825036	232,8723848	575,45488	2059,445444	1261,696056	106,255886
12	71552865	6,74473	36,65	3,64	0,503	2046,492977	1676,69272	792,927053	-2669,52929	-3055,608907	-993,9273632	-528,222717	-2136,809862	-361,360833	1263,98132	4226,977577	831,1837707	-1774,362094	1040,656365	182,7515237	530,093286	1676,446744	907,5961794	67,5444008
13	71552935	7,14267	36,06	4,06	0,569	2013,990346	1870,15727	896,969172	-2585,40713	-3354,050081	-1106,486334	-657,152819	-2696,092639	-462,41258	1204,70866	4566,136032	910,6166521	-2172,39441	1292,181152	230,1424304	735,573803	2359,300951	1295,405903	97,7737332
14	55224596	4,69897	19,86	0,999	0,74	1108,887012	460,169239	1166,53284	-783,770236	-454,4006144	-792,3105154	-39,7873747	-862,7673838	-782,111277	201,08134	340,6027302	359,0168778	-72,41816202	227,6733628	214,3213884	10,9583361	185,7726197	539,1184974	215,070358
15	63461798	3,82391	20,88	1,355	0,739	1166,017925	624,153472	1164,95645	-866,611858	-648,0830262	-832,0052537	-73,1969354	-1168,638647	-779,998891	233,789801	510,8081197	396,4271997	-140,0919054	324,2773968	224,7547513	27,3442514	341,3045186	729,2618205	214,199629
16	71552862	5,72125	31,27	1,805	0,772	1746,262489	831,436914	1216,97751	-1943,71785	-1292,923581	-1301,676993	-129,887907	-1626,263884	-851,215864	785,305168	1526,174875	928,8485404	-372,3001083	675,8211035	367,3321186	64,6367887	632,6896385	1060,149498	244,195299
17	71718729	5,01323	28,67	0,999	0,805	1601,061732	460,169239	1268,99856	-1633,91827	-656,0843682	-1244,458335	-39,7873747	-938,5510054	-925,543572	605,249044	710,0512329	814,1802951	-104,560651	357,5997144	366,1968598	10,9583361	202,090485	637,9880648	276,868176
18	71552776	4,74473	22,38	1,419	0,865	1250,066825	653,633784	1363,58231	-996,048837	-727,6151786	-1044,060681	-80,2747753	-1432,501263	-1068,65451	288,077653	614,8326159	533,3239898	-164,7127604	426,1470144	330,1263746	31,4047499	438,1268889	1046,332983	343,50552
19	71552778	5,92082	36,34	2,304	0,61	2029,627351	1061,29122	961,601397	-2625,71017	-1918,160336	-1195,425581	-211,630987	-1640,24537	-531,452896	1232,98786	2631,619715	991,4504195	-705,0346818	792,2392117	266,5574067	134,429808	814,5425836	844,8847087	120,468889
20	627458	4,00000	20,87	1,097	0,612	1165,403614	505,310966	964,754188	-865,698958	-524,4077643	-688,6589343	-47,9763896	-783,5280712	-534,943546	233,420482	413,111602	327,9538215	-91,77382668	217,3012016	154,0614506	14,5100208	185,2608646	404,91585	121,657718
21	71552936	5,82391	35,34	3,543	0,587	1973,445827	1632,01163	925,344295	-2482,35901	-2868,022053	-1118,509594	-500,445297	-2427,201122	-492,131666	1133,406	3825,864756	901,9803408	-1621,051225	1139,887996	240,0027342	488,834231	1853,529127	1203,102585	107,34942
22	71552939	6,69897	33,12	2,69	0,563	1849,522566	1239,09435	887,510797	-2180,38629	-2040,788693	-1005,412852	-288,482098	-1767,490896	-452,711887	933,015339	2551,406114	759,8646066	-875,7767661	777,9434467	206,9146315	213,946405	1024,783122	840,2806737	94,7132159
23	71552690	4,13668	21,27	1,258	0,721	1188,077271	579,472375	1136,58132	-899,712073	-613,0719604	-827,0968286	-63,0921882	-1058,552535	-742,464405	247,31129	492,3546674	401,5440474	-123,0368474	299,2873155	217,986699	21,8821543	287,0222445	644,4755945	198,925836
24	71552691	5,14267	24,67	0,999	0,586	1377,675952	460,169239	923,767899	-1209,78537	-564,545163	-779,5089339	-39,7873747	-683,2184959	-490,456326	385,612509	525,7359637	438,8340557	-89,9719802	223,9947809	166,9771666	10,9583361	147,1118313	338,0773111	106,80172
25	71552861	5,69897	30,53	1,612	0,784	1704,991966	742,535349	1235,89425	-1852,92929	-1127,388238	-1290,668774	-103,596333	-1474,951062	-877,884202	730,931858	1299,324879	899,2268975	-289,9223103	598,4545242	369,8871466	46,0408428	512,466106	976,4555272	255,760567
26	55193760	5,63827	21,26	0,999	0,775	1187,46296	460,169239	1221,70669	-898,781898	-486,5995299	-888,583363	-39,7873747	-903,5739493	-857,844385	246,927862	390,5833041	431,1718493	-77,54972699	255,3377223	251,7319005	10,9583361	194,5591625	591,3222197	247,053213
27	71552777	6,13668	29,79	1,419	0,809	1663,721443	653,633784	1275,30415	-1764,31206	-968,38741	-1299,587541	-80,2747753	-1339,761297	-934,764373	679,128135	1089,059648	883,5239006	-219,2172019	530,4436425	384,3195128	31,4047499	409,7626047	915,2394763	281,015945
28	44405271	5,12494	30,36	1,323	0,768	1695,49807	609,413317	1210,67192	-1832,35144	-920,1174539	-1257,288442	-69,780487	-1185,816734	-842,417826	718,789592	1054,538614	871,0926958	-194,198653	478,4604909	352,9673365	25,4523382	338,1426234	769,0199199	240,419151
29	44405274	4,16494	24,66	1,323	0,441	1376,949948	609,413317	695,190518	-1208,51065	-747,2469019	-586,3178914	-69,780487	-680,918203	-277,768048	385,003203	695,5113078	329,9008617	-157,7128454	223,1229817	94,51703965	25,4523382	194,1678345	253,5667641	45,5198861
30	44405275	4,68403	27,36	1,323	0,363	1527,958735	609,413317	572,231651	-1488,11819	-829,1967568	-535,5433785	-69,780487	-560,4836909	-188,199454	526,071479	856,4285541	334,3785644	-175,0090628	203,8007661	71,06236444	25,4523382	159,8252243	171,8020729	25,3866429
31	44405276	4,93181	29,06	1,323	0,42	1623,065231	609,413317	662,086207	-1679,13696	-880,8094058	-658,2057445	-69,780487	-648,4935266	-251,943808	630,547494	966,361983	436,5457775	-185,9023535	250,4798685	101,0530284	25,4523382	184,9217472	229,9925298	39,3217891
32	44405287	4,25885	29,56	1,594	0,691	1650,709221	734,244011	1089,28945	-1736,82207	-1079,307064	-1101,349168	-101,295684	-1285,472751	-681,963613	663,31774	1204,307844	742,8955462	-274,4583427	504,9689321	278,1899587	44,5156895	441,6453791	750,06641	175,113465
33	44405289	4,46471	34,26	1,594	0,831	1913,410899	734,244011	1309,98485	-2333,6228	-1251,073098	-1535,273206	-101,295684	-1545,915856	-986,295736	1033,08156	1618,127903	1200,400211	-318,1369422	703,9232367	466,3640539	44,5156895	531,1249059	1084,789992	304,570944
34	44405291	4,19723	28,08	1,582	0,334	1567,888943	728,716453	526,516174	-1566,91258	-1017,437878	-505,6362104	-99,7762704	-616,6652225	-159,330178	568,40223	1078,313686	323,9556915	-256,7778043	230,0890553	61,73379977	43,517866	210,2705421	173,9220789	19,7753782
35	11674015	4,28233	22,95	1,323	0,646	1281,843452	609,413317	1018,35164	-1047,33147	-695,6342529	-799,546839	-69,780487	-997,4448053	-596,032778	310,609581	602,7508977	418,8042199	-146,8195547	304,2671516	188,8055703	25,4523382	284,4272587	544,1018285	143,081325
36	44405293	4,24260	28,66	1,323	0,683	1600,335728	609,413317	1076,67828	-1632,4368	-868,4744979	-1055,378416	-69,780487	-1054,573997	-666,264257	604,426064	939,4855172	690,1626592	-183,2989657	401,6237311	263,4919104	25,4523382	300,7179841	608,2142019	169,101519
37	44405298	4,50585	21,96	1,582	0,686	1226,611318	728,716453	1081,40747	-959,020981	-795,9752642	-812,470286	-99,7762704	-1266,5639	-672,130092	272,164065	659,9764785	407,236413	-200,8857593	369,7134753	203,7370328	43,517866	431,8730296	733,6856347	171,339599
38	6914565	4,10902	22,95	1,323	0,561	1281,843452	609,413317	884,358006	-1047,33147	-695,6342529	-694,3433076	-69,780487	-866,2020677	-449,501174	310,609581	602,7508977	363,6984015	-146,8195547	264,2320001	142,3886884	25,4523382	247,0026194	410,3371823	93,7074203
39	21272512	4,18509	19,14	1,162	0,859	1068,900957	535,251908	1354,12393	-728,264441	-509,4830254	-886,5577554	-53,8302707	-1164,919198	-1053,88066	180,103552	368,1197372	387,236823	-94,44499482	296,3223951	278,3803468	17,2451326	291,7591125	844,9825902	336,406896
40	6852143	5,67778	20,26	1,582	0,251	1131,504822	728,716453	395,675329	-816,069209	-734,2585516	-274,2246772	-99,7762704	-463,4220684	-89,9813596	213,638297	561,6003123	126,7929459	-185,3098875	124,7855585	25,16046307	43,517866	158,0176829	98,22210276	8,39279399
41	6852148	3,92082	19,54	1,065	1,197	1090,960303	490,57081	1886,94569	-758,633593	-476,5897315	-1260,896682	-45,2182263	-1487,785319	-2046,41358	191,485867	351,4597107	562,1090781	-80,97247026	386,2606116	551,7114876	13,2769089	341,5170381	1503,81085	910,265011
42	11659169	5,40894	19,83	1,065	0,596	1107,490851	490,57081	939,531856	-781,797843	-483,8111577	-637,3277231	-45,2182263	-740,7853383	-507,338275	200,322772	362,1912425	288,4264645	-82,19938867	195,2377222	138,8504989	13,2769089	170,045242	372,8184809	112,36322
43	44405311	4,63639	21,54	1,065	1,105	1202,653193	490,57081	1741,91728	-921,923724	-525,3831517	-1283,15491	-45,2182263	-1373,435904	-1743,93247	256,525833	427,1087496	630,5966335	-89,26245954	393,0791533	518,2982501	13,2769089	315,2684437	1281,532041	716,097624
44	11660481	4,44612	28,65	1,065	0,748	1599,721417	490,57081	1179,14401	-1631,18377	-698,8437604	-1155,373389	-45,2182263	-929,7104582	-799,113199	603,730279	755,6946877	755,2641467	-118,7333714	353,9348133	315,9092429	13,2769089	213,4124849	587,2298301	222,121293
45	44405314	4,57349	30,05	1,226	0,713	1678,241519	564,732219	1123,97016	-1795,24239	-843,9778644	-1155,368158	-59,9232346	-1020,179308	-726,079488	697,064978	957,4308907	792,3316503	-165,0686603	407,4386072	301,1260301	20,2544084	269,5811238	614,2212466	192,377372
46	44405327	3,95861	20,86	1,421	0,653	1164,67761	654,555044	1029,38641	-864,620696	-678,8689254	-734,3368317	-80,5012203	-1082,93844	-609,019882	232,984517	534,4579907	349,4887395	-153,8945031	300,1516281	175,2858533	31,5377268	331,6808108	597,1394252	147,783158
47	44405330	3,92082	22,46	1,548	0,52	1254,143615	713,055037	819,725781	-1002,55618	-796,3506757	-629,6903222	-95,5336169	-939,4438131	-386,199578	290,905341	675,1084481	322,7055535	-196,6610752	280,3814575	119,6929957	40,7719067	313,4471068	412,5085446	74,6269048
48	44405331	4,01637	21,25	1,323	0,763	1186,736956	609,413317	1202,78994	-897,68322	-644,0216039	-874,2897932	-69,780487	-1178,096573	-831,48455	246,47523	516,626669	423,9767334	-135,926264	332,7105457	243,8475075	25,4523382	335,941174	759,039235	235,753969
49	44405336	4,62893	34,26	1,594	0,804	1913,410899	734,244011	1267,42217	-2333,6228	-1251,073098	-1485,390683	-101,295684	-1495,687543	-923,245513	1033,08156	1618,127903	1161,398038	-318,1369422	681,0520846	436,551132	44,5156895	513,86814	1015,443397	275,83765
50	44405338	3,92082	24,74	2,672	0,741	1381,585203	1230,80301	1168,10924	-1216,66081	-1514,259308	-988,49001	-284,634288	-2310,74048	-784,226519	388,904439	1414,164215	558,0615575	-645,4755642	759,7313582	267,7496224	209,680245	1330,791827	1445,866432	215,943443
51	6914566	4,17522	29,18	1,743	0,519	1629,822651	802,877862	818,149385	-1693,1478	-1165,262744	-816,7405354	-121,118109	-1055,750402	-384,715624	638,455935	1283,766767	543,9470288	-324,0144274	409,4801103	154,94938	58,2023224	396,6259646	462,6871179	74,1971924
52	44405380	5,34679	29,36	1,323	0,771	1639,651625	609,413317	1215,40111	-1713,63112	-889,8105548	-1220,625294	-69,780487	-1190,448831	-849,01207	650,076747	986,213754	817,8357637	-187,8021229	464,5083481	344,0132085	25,4523382	339,463493	775,0396224	243,247582
53	44410066	6,60206	32,79	1,258	0,842	1831,260778	579,472375	1327,32521	-2137,54153	-944,9676908	-1488,807982	-63,0921882	-1236,201435	-1012,57987	905,650147	1169,739275	1114,091938	-189,6446961	538,729359	458,2359465	21,8821543	335,1910261	878,9418214	316,826626
54	44410081	6,49485	33,02	0,872	0,88	1843,826228	401,669246	1387,22824	-2166,97624	-659,5118425	-1566,675544	-30,3142814	-895,562021	-1106,03903	924,421092	821,9861595	1180,405438	-91,74495317	392,9586589	503,9646919	7,2878195	168,3195848	665,4832585	361,687429
55	44410086	6,52288	32,61	0,872	0,842	1821,096725	401,669246	1327,32521	-2113,87937	-651,381805	-1480,544646	-30,3142814	-856,8900247	-1012,57987	890,65379	801,8452388	1101,759162	-90,61398043	371,355027	455,692598	7,2878195	161,0512391	609,2506107	316,826626
56	11948630	6,85387	36,2	1,258	0,734	2021,529616	579,472375	1157,07447	-2604,80004	-1043,150269	-1432,691128	-63,0921882	-1077,63878	-769,479808	1218,28867	1425,439866	1183,490644	-209,3488673	518,4233174	384,4032103	21,8821543	292,1974028	667,9255645	209,881219
57	44410094	6,38722	34,49	1,258	0,712	1926,367274	579,472375	1122,39376	-2365,33336	-994,0445709	-1324,327778	-63,0921882	-1045,33898	-724,044227	1054,21006	1294,395122	1042,477594	-199,49389	479,2117342	344,6782321	21,8821543	283,4394425	628,4864714	191,569065
58	44410097	5,45593	34,51	1,516	0,744	1927,037431	698,314881	1172,83842	-2366,97938	-1198,327365	-1384,32955	-91,6247305	-1316,341725	-790,589386	1055,31068	1560,944801	1090,088578	-289,8129437	603,6566717	376,4877063	38,2953248	430,1207034	826,9902276	218,576871
59	44410098	6,23657	22,94	1,065	1,057	1281,229141	490,57081	1666,25029	-1046,32786	-559,7093228	-1307,609896	-45,2182263	-1313,77534	-1595,7141	310,163226	484,7426882	684,6004115	-95,09446699	400,5706457	505,2328268	13,2769089	301,5735248	1172,613494	626,773116
60	44410128	6,34679	32,61	0,872	0,751	1821,096725	401,669246	1183,87319	-2113,87937	-651,381805	-1320,533289	-30,3142814	-764,2807702	-805,536052	890,65379	801,8452388	982,6854284	-90,61398043	331,2204575	362,5164056	7,2878195	143,6454639	484,6761665	224,804608
61	44410129	5,56864	21,24	1,065	0,798	1186,066799	490,57081	1257,96379	-896,669653	-518,1373288	-913,8784655	-45,2182263	-991,8568792	-909,517146	246,057908	415,4090455	442,9245391	-88,03139613	279,9557331	266,5813172	13,2769089	227,6780254	668,3603776	269,708151
62	44410130	6,11351	30,35	1,065	0,847	1694,827913	490,57081	1335,20719	-1830,90322	-740,3913577	-1386,070823	-45,2182263	-1052,760372	-1024,64147	717,937609	848,2207002	959,9379886	-125,7922973	424,6062119	429,1481122	13,2769089	241,658255	752,9597021	322,504389
63	44410133	5,06550	30,36	1,323	0,759	1695,49807	609,413317	1196,48436	-1832,35144	-920,1174539	-1242,554593	-69,780487	-1171,920445	-822,789347	718,789592	1054,538614	860,8845783	-194,198653	472,853532	344,7431372	25,4523382	334,1800145	751,1016244	232,065578
64	44410134	6,74473	24,65	1,065	1,075	1376,335637	490,57081	1694,62541	-1207,43256	-601,25692	-1428,595319	-45,2182263	-1336,148052	-1650,52473	384,488138	559,3792613	803,4627131	-102,1533929	437,6330824	561,3788693	13,2769089	306,7091194	1212,89119	659,342076
65	44410138	6,30103	27,35	1,065	0,987	1527,288578	490,57081	1555,90259	-1486,81311	-667,2012274	-1455,508211	-45,2182263	-1226,770351	-1391,35968	525,379584	688,8106583	908,3807302	-113,3573134	445,877525	525,1340595	13,2769089	281,6017682	1022,443265	510,313264
66	44410170	4,95861	31,26	1,594	0,755	1745,871564	734,244011	1190,17878	-1942,84769	-1141,528433	-1272,72814	-101,295684	-1404,532456	-814,139847	784,777882	1347,165471	907,9879538	-290,2806925	583,5462432	351,2537524	44,5156895	482,5503056	895,4421336	228,415859
67	44410210	6,63827	37,76	1,065	0,91	2108,482531	490,57081	1434,52012	-2833,70207	-921,0977893	-1852,626206	-45,2182263	-1131,064862	-1182,73621	1382,356	1312,797271	1596,20975	-156,4942726	567,5298709	616,2650943	13,2769089	259,632836	869,1359173	399,953568
68	44410211	5,58503	33,35	1,065	0,756	1862,367248	490,57081	1191,75517	-2210,77642	-813,5814879	-1359,450893	-45,2182263	-939,6538855	-816,297937	952,589646	1024,208325	1034,572663	-138,2272812	416,4515148	375,6848607	13,2769089	215,6949714	599,8580674	229,324674
69	44410215	5,67778	44,98	1,226	0,831	2511,693864	564,732219	1309,98485	-4021,12508	-1263,116184	-2015,320543	-59,9232346	-1189,016837	-986,295736	2336,73786	2144,528995	2068,440275	-247,0454559	710,6993464	612,1861923	20,2544084	314,1962327	834,3491407	304,570944
70	44410233	6,13668	29,05	1,065	0,95	1622,45092	490,57081	1497,57595	-1677,86614	-708,7732214	-1488,235215	-45,2182263	-1180,781999	-1288,99822	629,831801	777,3216916	986,6776572	-120,4203842	455,903051	516,8131512	13,2769089	271,0452683	947,2227574	455,046957
71	44410234	4,85387	35,87	1,226	0,829	2002,93275	564,732219	1306,83206	-2557,09527	-1007,26319	-1603,23543	-59,9232346	-1186,155184	-981,553937	1184,97444	1363,738963	1312,187265	-197,0046754	565,3782353	485,8365752	20,2544084	313,4400444	830,3378525	302,377164
72	44410238	6,20066	37,57	1,226	0,823	2098,095092	564,732219	1297,37369	-2805,85038	-1055,119776	-1667,252612	-59,9232346	-1177,570225	-967,397094	1362,02596	1496,403961	1429,416278	-206,3646633	587,9537853	501,5793129	20,2544084	311,1714796	818,3619827	295,859081
73	44410251	4,08092	18,8	0,872	0,92	1049,68978	401,669246	1450,28407	-702,321739	-375,4599162	-932,4492408	-30,3142814	-936,2693856	-1208,87324	170,566127	266,4075116	399,961619	-52,23037738	233,8799534	313,5821991	7,2878195	175,9704751	727,356702	413,284274
74	44410252	5,01323	20,5	0,872	0,846	1144,796276	401,669246	1333,63079	-835,354063	-409,4782306	-935,1364265	-30,3142814	-860,9607611	-1022,22344	221,255719	316,8698689	437,456961	-56,96267855	234,5539621	289,1902934	7,2878195	161,8163282	615,0529647	321,363455
75	44410256	6,49485	31,09	1,258	0,831	1736,154282	579,472375	1309,98485	-1921,28063	-895,8908106	-1393,046916	-63,0921882	-1220,051535	-986,295736	771,746783	1051,393568	988,294112	-179,7955022	504,0779474	423,1605191	21,8821543	330,812046	856,126606	304,570944
76	44410284	6,30980	29,61	0,872	0,908	1653,55739	401,669246	1431,36732	-1742,82074	-591,4552382	-1449,711301	-30,3142814	-924,0571762	-1177,54308	666,757176	661,0937833	979,5642937	-82,27757204	363,621307	481,1779831	7,2878195	173,675208	708,5059262	397,322303
77	44410286	6,48149	30,35	1,065	0,886	1694,827913	490,57081	1396,68662	-1830,90322	-740,3913577	-1449,892266	-45,2182263	-1101,23458	-1121,1728	717,937609	848,2207002	1004,138203	-125,7922973	444,1571473	469,578095	13,2769089	252,7853765	823,8959287	369,136138
78	44410287	6,10791	31,31	0,872	0,872	1748,719732	401,669246	1374,61708	-1949,19188	-625,4935282	-1472,35691	-30,3142814	-887,4205481	-1086,02064	788,624949	739,3753189	1052,120442	-87,012652	369,3013523	469,3188073	7,2878195	166,7894068	653,4385616	351,91263
79	44410291	6,67778	33,35	1,065	0,711	1862,367248	490,57081	1120,81737	-2210,77642	-813,5814879	-1278,531197	-45,2182263	-883,7221066	-722,011824	952,589646	1024,208325	972,9909573	-138,2272812	391,6627342	332,2915555	13,2769089	202,855985	530,5717402	190,763025
80	11495355	6,74473	33,77	1,323	0,761	1885,766908	609,413317	1199,63715	-2266,6799	-1023,373059	-1385,635721	-69,780487	-1175,008509	-827,131236	988,949037	1304,499474	1067,74914	-215,9916309	527,3029838	385,4535961	25,4523382	335,0605943	755,0652145	233,904926
81	44410295	4,72125	32,05	1,065	0,816	1789,990255	490,57081	1286,33892	-2042,28105	-781,9633516	-1410,318584	-45,2182263	-1014,229591	-951,010749	845,788704	946,1478008	1031,573185	-132,8553682	432,0342234	420,6740881	13,2769089	232,8136199	698,8520292	288,373849
82	23656593	4,00000	20,86	1,421	0,652	1164,67761	654,555044	1027,81002	-864,620696	-678,8689254	-733,212273	-80,5012203	-1081,280035	-607,156012	232,984517	534,4579907	348,9535346	-153,8945031	299,6919778	174,7494011	31,5377268	331,1728769	595,3119146	147,105254
83	25023956	4,00000	22,46	1,548	0,519	1254,143615	713,055037	818,149385	-1002,55618	-796,3506757	-628,4793793	-95,5336169	-937,6371904	-384,715624	290,905341	675,1084481	322,0849659	-196,6610752	279,8422624	119,2330807	40,7719067	312,8443239	410,9234988	74,1971924
84	25023957	4,39794	21,25	1,323	0,762	1186,736956	609,413317	1201,21355	-897,68322	-644,0216039	-873,1439351	-69,780487	-1176,552541	-829,306465	246,47523	516,626669	423,4210627	-135,926264	332,2744899	243,2087454	25,4523382	335,5008841	757,0509209	234,828234
85	25023955	4,72125	19,83	1,065	0,594	1107,490851	490,57081	936,379065	-781,797843	-483,8111577	-635,1890395	-45,2182263	-738,2994815	-503,939033	200,322772	362,1912425	287,4585905	-82,19938867	194,582562	137,9201799	13,2769089	169,4746204	370,3205416	111,235839
86	22947105	5,00000	20,13	0,904	1,376	1124,077245	416,409402	2169,12053	-805,390469	-416,8220755	-1493,451054	-32,5800067	-1451,721721	-2704,21972	209,458674	316,7151121	685,9923919	-60,11214735	388,3387588	751,1865478	8,11995167	282,8618022	1686,788391	1382,74055
87	25023954	5,63827	25,83	0,904	0,742	1442,625367	416,409402	1169,68563	-1326,54291	-534,9437528	-1033,555669	-32,5800067	-782,8324981	-786,344618	442,762238	521,6552744	609,2837382	-77,14710803	268,7531838	280,3342447	8,11995167	152,5315823	490,4915685	216,818889
88	25023973	5,13077	27,61	1,033	0,649	1541,8645	475,830655	1023,08083	-1515,3278	-653,3300827	-966,2006149	-42,5417108	-782,4228569	-601,581541	540,565729	680,9273579	608,7593827	-107,66538	287,0905816	229,2188772	12,1157169	174,2064975	428,7903672	145,083989
89	16220130	5,12494	26,94	1,065	0,787	1504,614921	490,57081	1240,62344	-1442,99525	-657,2961631	-1143,342902	-45,2182263	-978,1846666	-884,615557	502,326366	668,510726	702,9655037	-111,6744455	350,2494177	328,919489	13,2769089	224,5396065	650,0613984	258,707843
90	25023972	5,16115	26,94	1,065	0,836	1504,614921	490,57081	1317,86683	-1442,99525	-657,2961631	-1214,529436	-45,2182263	-1039,088159	-998,200224	502,326366	668,510726	746,7333687	-111,6744455	372,0565606	371,1527623	13,2769089	238,5198361	733,5293033	310,10176
91	25023971	4,95861	25,91	1,033	0,703	1446,702158	475,830655	1108,2062	-1334,051	-613,0072003	-981,9985751	-42,5417108	-847,5242964	-705,855427	446,526531	599,468853	580,5266158	-101,0203799	291,784685	252,3507252	12,1157169	188,701337	503,1138544	184,395948
92	16220129	5,09151	25,91	1,033	0,523	1446,702158	475,830655	824,454968	-1334,051	-613,0072003	-730,5622401	-42,5417108	-630,5194979	-390,668582	446,526531	599,468853	431,8853771	-101,0203799	217,0745238	139,6681192	12,1157169	140,3852052	278,4575544	75,9259903
93	25023970	4,63827	25,24	1,065	0,745	1409,452579	490,57081	1174,41482	-1266,23732	-615,7241691	-1013,872036	-45,2182263	-925,9816729	-792,716054	412,915596	586,6223268	583,9366882	-104,6113747	310,5875669	276,1072382	13,2769089	212,5565525	582,5288764	219,459415
94	16220126	5,19382	30,94	0,904	0,896	1728,000701	416,409402	1412,45058	-1903,27701	-640,76454	-1494,955714	-32,5800067	-945,3071675	-1146,62426	760,924598	748,4526017	1055,612176	-92,40809138	388,7300122	489,6375448	8,11995167	184,1890805	715,2201725	381,776707
95	25023969	4,95861	27,54	0,904	0,78	1537,731863	416,409402	1229,58867	-1507,21567	-570,2104457	-1158,114821	-32,5800067	-822,9236503	-868,949052	536,230757	592,7037847	727,7201192	-82,23310698	301,1420233	330,2057235	8,11995167	160,3431728	542,0170412	251,865804
96	16220128	5,25181	29,24	0,904	0,84	1632,894205	416,409402	1324,17241	-1699,53561	-605,4978471	-1324,383427	-32,5800067	-886,2254695	-1007,77523	642,072433	668,3324825	883,6981266	-87,32209243	344,3764794	406,6599452	8,11995167	172,677263	628,6114797	314,574313
97	25023967	5,53760	27,54	0,904	0,712	1537,731863	416,409402	1122,39376	-1507,21567	-570,2104457	-1057,150965	-32,5800067	-751,1815884	-724,044227	536,230757	592,7037847	664,2778524	-82,23310698	274,8886162	275,1410425	8,11995167	146,3645372	451,6309778	191,569065
98	25023966	5,45593	27,54	0,904	0,54	1537,731863	416,409402	851,253695	-1507,21567	-570,2104457	-801,771799	-32,5800067	-569,7163733	-416,47854	536,230757	592,7037847	503,8062364	-82,23310698	208,4829392	158,2642817	8,11995167	111,0068119	259,7833156	83,573132
99	25023968	5,67778	26,13	1,263	0,698	1459,100069	581,775525	1100,32422	-1357,014	-755,9177676	-983,3698843	-63,5947126	-1028,857633	-695,850516	458,105087	745,5581971	586,3192199	-152,3073998	357,2494497	250,9058001	22,1441087	280,0793778	606,4143937	180,489385
100	25023964	5,46852	29,65	0,904	0,679	1655,623708	416,409402	1070,3727	-1747,17919	-613,9262347	-1085,44498	-32,5800067	-716,3655879	-658,483136	669,25988	687,0680403	734,3474658	-88,53759541	282,2458461	269,4113966	8,11995167	139,5807876	410,736487	166,147848
101	15942656	5,79588	27,54	0,904	0,579	1537,731863	416,409402	912,733129	-1507,21567	-570,2104457	-859,6775401	-32,5800067	-610,8625558	-478,808923	536,230757	592,7037847	540,1922424	-82,23310698	223,5400404	181,9501922	8,11995167	119,0239706	298,6626149	103,019892
102	25023965	5,58503	28,2	0,872	0,512	1574,981442	401,669246	807,114615	-1581,12081	-563,3496784	-778,6132772	-30,3142814	-521,0542668	-374,407923	576,150845	599,7571164	501,1057604	-78,3677965	195,2943164	145,7236835	7,2878195	97,93139482	225,2743328	71,2353039
103	25023962	5,69897	26,94	0,904	0,697	1504,614921	416,409402	1098,74783	-1442,99525	-557,9302643	-1012,592126	-32,5800067	-735,3561336	-693,858103	502,326366	567,4494801	622,5755478	-80,46211615	263,3020803	257,9916787	8,11995167	143,2810146	432,8020328	179,714754
104	25023963	5,13077	26,94	0,904	0,669	1504,614921	416,409402	1054,60874	-1442,99525	-557,9302643	-971,9141064	-32,5800067	-705,8152847	-639,230287	502,326366	567,4494801	597,5653393	-80,46211615	252,7246653	237,6798571	8,11995167	137,5251059	398,7272994	158,914583
105	25023961	5,69897	25,24	0,904	0,816	1409,508425	416,409402	1286,33892	-1266,33767	-522,6635714	-1110,540083	-32,5800067	-860,9047418	-951,010749	412,96468	497,9799143	639,6377158	-75,3761172	288,77127	331,2552512	8,11995167	167,7436269	593,2039759	288,373849
106	25023959	5,60206	23,54	0,904	0,972	1314,346083	416,409402	1532,25665	-1101,11745	-487,3761699	-1233,537633	-32,5800067	-1025,489472	-1349,39047	334,841351	433,0080238	662,5128221	-70,28713175	320,7540495	438,2854394	8,11995167	199,8122615	841,6979425	487,398506
107	25023960	5,45593	25,24	0,904	0,805	1409,508425	416,409402	1268,99856	-1266,33767	-522,6635714	-1095,569567	-32,5800067	-849,2994083	-925,543572	412,96468	497,9799143	631,0151486	-75,3761172	284,8785201	322,3845458	8,11995167	165,482377	577,3185291	276,868176
108	25023958	[illegible]	[illegible]	0,904	1,162	1219,239587	416,409402	[illegible]	[illegible]	[illegible]	[illegible]	[illegible]	[illegible]	[illegible]	[illegible]	[illegible]	[illegible]	[illegible]	[illegible]	[illegible]	[illegible]	[illegible]	[illegible]	[illegible]

	0,000042 x	-0,014 x	-0,017 x	0,095 x	-0,035 x	0,018 x	-0,317 x	-0,805 x	-7,912 x	-4,020 x	0,677 x	-12,668 x	-144,091 x	-206,850 x	-62,484 x				
N	[D143^{4}]	[D143^{3} x D312]	[D143^{3} x D470]	[D143^{2} x D312^{2}]	[D143^{2} x D312 x D470]	[D143^{2} x D470^{2}]	[D143 x D312^{3}]	[D143 x D312^{2} x D470]	[D143 x D312 x D470^{2}]	[D143 x D470^{3}]	[D312^{4}]	[D312^{3} x D470]	[D312^{2} x D470^{2}]	[D312 x D470^{3}]	[D470^{4}]	SUM of H to AO columns	Intercept		PEC50 FINAL
1	5,66492206	-104,7487717	-102,1540664	42,12611297	-12,07451336	4,714429725	-8,027422419	-15,88342052	-121,8738916	-48,32355723	0,98019944	-14,31538183	-127,0567747	-142,3257278	-33,5478347	747,7707881	-743,64	=	4,14
2	10,4968452	-225,199492	-146,8866203	105,0808235	-20,14419087	5,260384259	-23,23281365	-30,74523649	-157,7802336	-41,84166169	3,29149922	-32,15069403	-190,8505231	-142,9838717	-22,5411592	747,914197	-743,64	=	4,28
3	59,170746	-1456,873563	-391,0621895	780,1608076	-61,54880462	6,61449051	-197,9560029	-107,8087072	-227,6867344	-24,84867241	32,1859868	-129,3816121	-316,0710315	-97,45127495	-6,32245652	750,8266642	-743,64	=	7,19
4	6,1466131	-101,4119779	-108,9824362	36,39067225	-11,4939384	4,945257766	-6,1874711	-13,49092224	-114,0693121	-49,83994775	0,67413969	-10,84923003	-106,1095191	-130,9786384	-34,0206096	748,0657686	-743,64	=	4,43
5	13,6556958	-282,2640612	-176,3864311	126,8950813	-23,30590134	5,830802091	-27,03063638	-34,27102192	-168,4987048	-42,81023891	3,68961761	-34,52810233	-196,3678874	-140,9480012	-21,2883925	748,0181961	-743,64	=	4,38
6	32,1675618	-536,7027491	-317,8254091	194,7587346	-33,89715641	8,036572595	-33,48747083	-40,23447514	-187,4619197	-45,13457447	3,68961761	-32,72034829	-176,3440794	-119,9484235	-17,1681579	749,0803468	-743,64	=	5,45
7	11,7063792	-207,0353185	-190,2589578	79,63678874	-21,50928342	7,913697157	-14,51460828	-27,06245499	-195,6715976	-73,10885171	1,69515926	-23,32882928	-195,1110158	-205,9497793	-45,7442347	748,6766665	-743,64	=	5,04
8	73,2710452	-1500,47451	-460,6829735	668,3013005	-60,30556267	7,412824584	-141,0386916	-87,85631974	-212,229852	-26,49241245	19,0729487	-87,69465913	-245,0387984	-86,41462705	-6,4126149	750,3364823	-743,64	=	6,70
9	67,7109398	-2174,764028	-436,5086335	1519,192784	-89,62003604	7,201759615	-502,8470619	-204,7754133	-323,3839288	-26,39007517	106,652871	-320,5789575	-585,6037913	-135,0092247	-6,54965616	750,6873156	-743,64	=	7,05
10	62,7817046	-2073,469879	-413,176986	1489,394711	-87,22876588	6,9590656	-506,9255298	-204,9480324	-321,3231907	-26,03286465	110,558497	-329,9227647	-598,3273371	-136,9481295	-6,59582095	751,3293718	-743,64	=	7,69
11	59,653109	-2088,138871	-408,1004923	1589,765696	-91,31715878	7,145176172	-573,4943517	-227,4039912	-349,675938	-27,78529337	132,568021	-387,9969213	-690,1193281	-154,9211807	-7,31800833	750,8547939	-743,64	=	7,22
12	75,7370168	-2430,129328	-419,696716	1695,891361	-86,08267231	5,952161889	-560,7753939	-196,4971838	-267,0068725	-18,74865517	118,821075	-307,3133486	-483,0318629	-95,82102903	-3,99982855	750,8215788	-743,64	=	7,19
13	71,0389769	-2583,42236	-452,5027573	2043,344436	-105,1910823	7,376622334	-765,7905887	-272,1429183	-375,0444804	-26,70853649	183,904381	-482,3915103	-768,9775169	-154,709978	-6,54965616	751,1931443	-743,64	=	7,56
14	6,52854417	-106,1022529	-98,22689592	37,50423518	-10,20463924	3,782302679	-6,281426768	-11,79845967	-85,9391496	-32,34732437	0,67413969	-9,346251714	-78,74653592	-83,73675734	-18,7368385	748,6617297	-743,64	=	5,03
15	7,98156523	-167,3216748	-114,0504306	76,28932768	-15,28340219	4,17078284	-16,48153481	-22,79310941	-122,2385603	-33,87618139	2,2816295	-23,29011392	-144,4789276	-113,1170582	-18,6357634	747,825364	-743,64	=	4,19
16	40,1518223	-748,691683	-400,2051617	303,6324655	-47,7023101	10,20873424	-58,34661741	-63,27855561	-266,131425	-57,8385416	7,1845036	-57,51196522	-279,786049	-171,7847467	-22,1941561	749,5500623	-743,64	=	5,91
17	28,3726218	-319,3647265	-321,6302751	78,18471804	-23,1421326	9,330956454	-9,06941096	-18,53148633	-146,8385724	-60,12452051	0,67413969	-10,16720626	-93,18795461	-107,7974831	-26,239343	748,9774951	-743,64	=	5,34
18	10,5438709	-215,9132586	-164,4949641	96,16255499	-21,53231958	6,567754669	-20,29340654	-31,36818912	-188,028061	-58,24215821	2,74421114	-31,30924411	-217,0871155	-189,9705191	-34,9811325	748,0907242	-743,64	=	4,46
19	73,2710452	-1500,47451	-496,4957842	668,3013005	-64,99362761	8,610146299	-141,0386916	-94,68613965	-246,5092832	-33,1636039	19,0729487	-94,51191178	-284,6175407	-108,1751414	-8,65144747	749,978301	-743,64	=	6,34
20	7,96475833	-135,2486493	-94,30122062	49,95060122	-10,23614963	2,857418242	-8,741194481	-12,36563313	-67,83606692	-19,23032104	0,98019944	-10,23482906	-64,94610969	-52,01353678	-8,76546831	748,5080878	-743,64	=	4,87
21	65,4889453	-2121,016304	-439,1880742	1494,058031	-90,92546409	7,537804477	-498,6699584	-209,4985081	-341,3087257	-28,73395587	106,652871	-330,7202953	-623,2403308	-148,2316517	-7,41859804	749,8247553	-743,64	=	6,19
22	50,5249273	-1325,648578	-346,7560987	756,4819538	-58,15750923	6,09051905	-204,5460108	-108,5545194	-223,4104748	-23,75968763	35,4402733	-138,827187	-330,4900524	-99,29627489	-6,27773552	750,7593292	-743,64	=	7,12
23	8,60292051	-164,3281367	-117,7080425	68,26929227	-14,37246089	4,121716833	-13,43882041	-19,53064356	-110,0704357	-32,05578298	1,69515926	-18,18387666	-118,541073	-97,53083152	-16,8853666	747,9375984	-743,64	=	4,30
24	15,554479	-203,4716693	-149,1679972	57,8895102	-12,47335015	3,661066644	-7,804014753	-11,60782868	-66,95494001	-19,95702646	0,67413969	-7,401220952	-49,38138322	-41,58280943	-7,36817445	749,0519004	-743,64	=	5,42
25	36,4885418	-622,3422352	-378,2856644	230,8604257	-41,24313109	10,03679483	-40,57812623	-50,04305266	-239,3284495	-59,14614349	4,5703356	-41,60259912	-230,1437804	-160,6825507	-23,6066133	749,5834584	-743,64	=	5,95
26	8,58514129	-130,2935541	-126,3277404	43,00766492	-12,2555608	4,757313766	-6,726529884	-13,23207855	-100,9401144	-39,79065488	0,67413969	-9,78830416	-86,37169127	-96,18915009	-22,5411592	748,8319552	-743,64	=	5,20
27	33,0818369	-509,0043161	-362,6829788	170,3337715	-35,67122303	10,17598642	-27,00861661	-39,04534335	-218,8945155	-63,41355294	2,74421114	-29,28228727	-189,8885929	-155,4116073	-26,7647704	749,5203385	-743,64	=	5,89
28	35,6825891	-502,2835683	-364,4097008	153,776167	-32,79000769	9,524348699	-22,30752415	-32,83624805	-187,4366059	-55,28876225	2,07361115	-22,52942156	-148,7576026	-123,9649746	-21,7377363	749,209601	-743,64	=	5,57
29	15,5217175	-269,0367037	-112,0804488	101,4216659	-12,41825565	2,071246246	-18,11641355	-15,3126992	-50,1914803	-8,50138785	2,07361115	-12,93681629	-49,04942376	-23,47097356	-2,36332687	747,6712852	-743,64	=	4,04
30	23,5349707	-367,6139203	-126,060293	124,8871294	-12,58680706	1,72804381	-20,10322335	-13,98663555	-37,73632011	-5,26123146	2,07361115	-10,64867191	-33,23303315	-13,08986631	-1,08491555	748,2995291	-743,64	=	4,66
31	29,9647776	-440,6208002	-174,8211706	140,9179708	-16,43262476	2,610290106	-21,35453144	-17,19017401	-53,66229309	-8,656448573	2,07361115	-12,32077742	-44,48927325	-20,27510944	-1,94431487	748,7681525	-743,64	=	5,13
32	32,0589629	-558,4666925	-302,5705047	211,5887302	-33,69251061	7,308285368	-37,98481959	-41,75423954	-177,9876285	-39,20673047	4,36959524	-35,45294259	-174,8114081	-108,7872381	-14,2456288	748,9601888	-743,64	=	5,33
33	57,8762166	-869,7817167	-566,7122024	284,2941114	-54,44170051	14,20158383	-44,02990354	-58,20512427	-298,3825598	-79,04369292	4,36959524	-42,6358832	-252,8225014	-189,2112174	-29,7970679	748,6028719	-743,64	=	4,97
34	26,0932605	-474,9518667	-125,3225345	188,0261687	-14,58174152	1,54042932	-35,27030981	-18,88208908	-39,20031327	-4,205431909	4,23949237	-16,75232899	-40,22934246	-12,19274164	-0,7775983	748,0988038	-743,64	=	4,46
35	11,6575454	-217,051124	-132,4568113	87,89504854	-15,7647902	3,85170717	-16,86510546	-20,88153956	-100,2616152	-24,87645267	2,07361115	-18,95052908	-105,2499181	-73,77562372	-10,8817691	747,7810326	-743,64	=	4,15
36	28,3211944	-422,3673849	-272,5152541	136,9987593	-25,97936937	6,710916837	-21,0554813	-27,56302079	-139,9223789	-36,70532888	2,07361115	-20,0359309	-117,6516813	-87,19216208	-13,5972866	748,4150397	-743,64	=	4,78
37	9,77451079	-227,4178813	-123,2485529	115,0804727	-18,33033427	3,977227321	-27,59312858	-30,34026441	-129,3708721	-28,50593053	4,23949237	-34,4074781	-169,7064044	-105,6414413	-13,8377633	747,8279357	-743,64	=	4,19
38	11,6575454	-217,051124	-115,0282835	87,89504854	-13,6904757	2,904784701	-16,86510546	-18,13396856	-75,61281093	-16,29219054	2,07361115	-16,45703841	-79,37476512	-48,3174404	-6,18900567	747,7003744	-743,64	=	4,07
39	5,63659751	-110,5390565	-102,1274227	47,14785417	-12,80265606	4,735649017	-9,528628382	-17,86151142	-129,8388766	-48,7722583	1,23399231	-17,0734323	-143,5607194	-152,3495273	-34,0206096	747,7324306	-743,64	=	4,10
40	7,07771053	-178,5142682	-35,39808429	97,9265648	-5,707144564	0,453083773	-25,45366864	-10,24043508	-15,97662931	-1,288052252	4,23949237	-12,58932508	-22,71943064	-5,174675673	-0,24800722	749,3697333	-743,64	=	5,73
41	6,11649961	-107,7143784	-151,3065765	41,25644998	-17,03285735	9,57909421	-7,487421997	-21,33917542	-235,8422906	-134,6937074	0,87073574	-18,31691215	-234,1663889	-377,822268	-128,276185	747,6883835	-743,64	=	4,05
42	6,49572676	-112,6853029	-78,81402571	42,51618158	-8,739810504	2,447321701	-7,600873592	-10,78601306	-59,35497164	-16,87853832	0,87073574	-9,120200204	-58,05354935	-46,63842532	-7,88414267	748,4222312	-743,64	=	4,79
43	9,03293312	-144,3005748	-187,1200454	50,13658814	-19,1081463	9,920272932	-8,253986829	-21,71586943	-221,5590019	-116,8108409	0,87073574	-16,90909602	-199,5541729	-297,2295154	-93,1576549	748,8557026	-743,64	=	5,22
44	28,2777335	-339,6095642	-298,1065274	88,70797743	-22,88578315	8,042853563	-10,97912481	-19,5533193	-135,0429729	-48,19534133	0,87073574	-11,4461573	-91,44068137	-92,19553589	-19,5603142	748,7177256	-743,64	=	5,08
45	34,2519266	-451,3891135	-328,0875294	129,3792431	-27,63851957	8,042780741	-17,5711591	-25,91195918	-148,1831486	-43,79040464	1,52914906	-16,64447991	-110,102465	-91,92096	-16,1483277	748,7461272	-743,64	=	5,11
46	7,94492985	-174,8672521	-100,4308577	83,70942385	-14,13007826	3,249047522	-18,98726886	-22,12495436	-99,97720578	-23,3453924	2,75971512	-23,73584536	-124,0656516	-81,84442717	-11,3611465	747,7414537	-743,64	=	4,11
47	10,6820903	-237,8537475	-99,8577942	115,1890507	-14,21329264	2,389018308	-26,43228328	-22,51478725	-74,37033583	-12,69443015	3,88661719	-24,43574034	-93,36533122	-45,02321243	-4,56859658	747,45466	-743,64	=	3,82
48	8,56416504	-172,2346284	-124,1437183	75,33614022	-15,95949594	4,605494387	-15,61379737	-22,83358023	-129,4905914	-37,94757155	2,07361115	-22,38274564	-146,8269599	-121,5595125	-21,1771536	747,890782	-743,64	=	4,26
49	57,8762166	-869,7817167	-548,2991705	284,2941114	-52,6728366	13,29372932	-44,02990354	-56,31398305	-279,3080709	-71,58669248	4,36959524	-41,2506018	-236,6604979	-171,360987	-26,1092039	748,9603955	-743,64	=	5,33
50	15,7317796	-548,8664698	-190,2339553	416,4884838	-42,42636879	5,887216536	-149,7481873	-105,3038157	-287,1609427	-40,4658069	34,5011647	-179,0757828	-564,867211	-224,8777925	-18,8383242	747,6475951	-743,64	=	4,01
51	30,4669203	-587,7811606	-218,738493	246,6324494	-26,97561148	4,019144879	-49,03509828	-37,02352802	-108,4044922	-16,40205751	6,24708608	-34,81519308	-117,9143149	-50,40283355	-4,53355477	747,7513316	-743,64	=	4,12
52	31,2085431	-454,2676632	-330,8612755	143,812819	-30,78528968	8,976978214	-21,57275722	-31,87872694	-182,6816862	-54,09668162	2,07361115	-22,61742712	-149,9220413	-125,4233712	-22,0793837	749,0920748	-743,64	=	5,46
53	48,5587988	-601,7671132	-503,3840876	162,1946083	-39,87667831	13,35496696	-20,71412805	-35,15595396	-231,3821463	-78,69413679	1,69515926	-21,23553973	-161,66742	-155,3361036	-31,4063742	749,6951099	-743,64	=	6,06
54	49,9053509	-425,7686471	-537,0063827	79,00374712	-29,28633249	14,78848103	-6,946147205	-17,77505487	-176,3910905	-90,45319518	0,39133968	-7,391650362	-84,8467783	-122,9192758	-37,4714176	750,2468815	-743,64	=	6,61
55	47,4896768	-410,216148	-495,0487197	77,06793812	-27,33508683	13,20713014	-6,860519572	-16,79783822	-159,4955273	-78,25736045	0,39133968	-7,07246546	-77,67731318	-107,6733564	-31,4063742	749,9246836	-743,64	=	6,29
56	72,1086879	-809,5024979	-590,3005596	197,6497376	-42,36066529	12,36717721	-22,86633549	-33,83083913	-194,1009659	-57,54719975	1,69515926	-18,51174129	-122,8543234	-102,9021177	-18,1365081	750,7144341	-743,64	=	7,08
57	59,4598113	-700,4790376	-495,4890698	179,4792346	-37,31338701	10,56711461	-21,78991592	-31,27200213	-174,0421932	-50,05356099	1,69515926	-17,95689346	-115,6001271	-93,92389917	-16,0579244	750,1315986	-743,64	=	6,50
58	59,5425956	-845,0197904	-518,2987929	260,8275411	-47,01953097	11,54634207	-38,1471673	-47,47192122	-229,0920668	-57,13008429	3,57507162	-32,83823665	-183,3079565	-129,1437817	-19,145256	749,5341855	-743,64	=	5,90
59	11,6352144	-174,4726105	-216,4174231	56,90200572	-20,74455226	10,30200694	-8,793265188	-22,12974097	-215,9738737	-108,9200113	0,87073574	-16,17458325	-182,5938864	-260,1537326	-77,9954709	749,4886817	-743,64	=	5,85
60	47,4896768	-410,216148	-441,5458296	77,06793812	-24,38081973	10,50664717	-6,860519572	-14,9823949	-126,8832224	-55,52757815	0,39133968	-6,308101616	-61,79450905	-76,39972364	-19,8760089	750,1157435	-743,64	=	6,48
61	8,54483648	-138,4121715	-129,618547	48,76320667	-13,4213639	5,032019649	-8,140151951	-15,46630518	-113,9565695	-43,38840953	0,87073574	-12,21127477	-104,0739506	-111,9473387	-25,3385058	748,4805254	-743,64	=	4,85
62	35,6262074	-403,8533214	-401,4181863	99,56923604	-29,0877473	11,57540737	-11,63185476	-23,45759876	-183,44964	-74,13644325	0,87073574	-12,96108989	-117,2473616	-133,86139	-32,1590401	749,4559292	-743,64	=	5,82
63	35,6825891	-502,2835683	-360,1392746	153,776167	-32,40574978	9,302429747	-22,30752415	-32,45144827	-183,069301	-53,367706	2,07361115	-22,2654049	-145,2915251	-119,6577035	-20,7365517	749,1513805	-743,64	=	5,52
64	15,4940367	-216,2817626	-272,8464701	65,66329457	-24,34628137	12,29656716	-9,445995143	-24,17727523	-239,9748444	-123,0851489	0,87073574	-16,45002554	-188,8657407	-273,6720795	-83,4455713	750,4974525	-743,64	=	6,86
65	23,4937085	-295,5358336	-342,3081775	80,85672869	-27,52547503	12,76423409	-10,48200751	-24,6327439	-224,4811323	-105,7130175	0,87073574	-15,10341879	-159,2100809	-211,8149246	-59,297727	750,6324913	-743,64	=	7,00
66	40,1158802	-660,7275539	-391,1295567	236,6878476	-41,17993967	9,759705958	-40,17462041	-48,25154197	-224,7342884	-54,08901265	4,36959524	-38,73657259	-208,6928546	-141,9007411	-20,302861	749,6101817	-743,64	=	5,97
67	85,3389291	-777,6010827	-830,4016894	154,1040219	-48,36785959	20,67954821	-14,47082762	-31,35349324	-263,437276	-114,3799999	0,87073574	-13,9251379	-135,3377782	-166,008099	-42,848439	750,6525896	-743,64	=	7,02
68	51,9431714	-535,8494771	-475,3949695	120,2277196	-31,34930439	11,13505815	-12,78170201	-23,00708813	-160,5954925	-57,92774458	0,87073574	-11,5685761	-93,4070914	-95,18543214	-20,4106397	749,8478976	-743,64	=	6,21
69	171,843844	-1513,170309	-1281,852184	289,7938022	-72,15239605	24,47111197	-26,29739045	-45,19849648	-301,2548516	-103,7589776	1,52914906	-19,39910632	-149,5615751	-145,5288285	-29,7970679	750,1979779	-743,64	=	6,56
70	29,9194381	-354,2921577	-394,9800129	91,24668493	-29,89800456	13,34469322	-11,13512075	-25,18660948	-220,9241607	-100,1378567	0,87073574	-14,53723187	-147,4970955	-188,8756254	-50,8936802	750,4675072	-743,64	=	6,83
71	69,4916512	-767,3381677	-648,4708283	184,2843348	-45,77239013	15,48674131	-20,97067056	-35,95647908	-239,0786124	-82,14589525	1,52914906	-19,35241773	-148,8425301	-144,4806056	-29,5112468	749,663095	-743,64	=	6,03
72	83,6696267	-881,9890657	-739,966654	202,2115786	-49,86163277	16,74820444	-21,96701861	-37,3922211	-246,8255628	-84,19389107	1,52914906	-19,21235198	-146,6957909	-141,366162	-28,6661109	749,771354	-743,64	=	6,14
73	5,24216922	-78,55912164	-103,5875459	25,60528718	-9,923208231	5,238607254	-3,954439752	-10,57930373	-109,7559153	-58,8410425	0,39133968	-7,72763447	-92,73542504	-140,4544355	-44,7631608	747,6177737	-743,64	=	3,98
74	7,41617539	-101,9056671	-123,563954	30,45538748	-10,85348271	5,2688446	-4,31272933	-10,60979177	-101,2185814	-49,89938634	0,39133968	-7,106063871	-78,41709294	-109,215195	-32,0074361	748,9361698	-743,64	=	5,30
75	39,2301911	-512,7938593	-423,3531363	145,7849383	-35,37399835	11,69221861	-19,63834018	-32,89470086	-213,6711227	-71,7211583	1,69515926	-20,95811582	-157,4709226	-149,3272971	-29,7970679	749,6256582	-743,64	=	5,99
76	32,2807971	-307,0941404	-399,6506065	63,53986071	-24,30338312	12,66276332	-6,229357663	-16,44801186	-168,4155865	-89,11128899	0,39133968	-7,626839237	-90,33201733	-135,0297682	-42,4729894	749,5811443	-743,64	=	5,95
77	35,6262074	-403,8533214	-419,9014322	99,56923604	-30,42708868	12,66592486	-11,63185476	-24,53770071	-200,7324045	-84,8560245	0,87073574	-13,55788151	-128,2932188	-153,2167567	-38,5038647	749,8272558	-743,64	=	6,19
78	40,3782974	-363,2238387	-453,9561737	71,06375216	-26,10353026	13,06145741	-6,587857627	-16,70494249	-164,2647938	-83,46906968	0,39133968	-7,324453541	-83,31112173	-119,5973155	-36,1272892	749,5893616	-743,64	=	5,95
79	51,9431714	-535,8494771	-447,0976499	120,2277196	-29,48327437	9,848908433	-12,78170201	-21,6376186	-142,0459848	-48,18701622	0,87073574	-10,87997038	-82,61814873	-79,17970905	-15,9679013	750,0598392	-743,64	=	6,42
80	54,6033391	-691,0685081	-496,8044739	190,2262526	-40,19262549	11,56814122	-24,8108751	-36,18825777	-204,6878176	-59,82709126	2,07361115	-22,32407527	-146,0582336	-120,6061089	-20,9559842	749,6014272	-743,64	=	5,97
81	44,3271518	-475,7719513	-455,5950206	111,0645068	-31,25841512	11,98394751	-12,284968	-23,86796325	-179,8272154	-70,01273615	0,87073574	-12,48671706	-108,8219679	-119,6948806	-27,7032085	749,4787738	-743,64	=	5,84
82	7,94492985	-174,8672521	-100,2770585	83,70942385	-14,10843955	3,239104	-18,98726886	-22,09107235	-99,67123134	-23,23830361	2,75971512	-23,69949644	-123,6859558	-81,4689946	-11,2917127	747,7405873	-743,64	=	4,11
83	10,6820903	-237,8537475	-99,66575998	115,1890507	-14,18595938	2,379838611	-26,43228328	-22,47148958	-74,08457112	-12,6213338	3,88661719	-24,38874853	-93,00657908	-44,76396232	-4,53355477	747,4549283	-743,64	=	3,82
84	8,56416504	-172,2346284	-123,9810135	75,33614022	-15,93857917	4,593430228	-15,61379737	-22,80365418	-129,151389	-37,79856293	2,07361115	-22,35341046	-146,4423445	-121,0821851	-21,0663512	747,8893102	-743,64	=	4,25
85	6,49572676	-112,6853029	-78,54954912	42,51618158	-8,710482281	2,430924282	-7,600873592	-10,74981839	-58,95728451	-16,7091897	0,87073574	-9,089595505	-57,66458194	-46,17048476	-7,77884674	748,4173727	-743,64	=	4,78
86	6,89369056	-100,0124639	-190,2583443	31,55759637	-17,64432431	13,43839579	-4,7181961	-18,21070549	-272,5688878	-210,8178431	0,45202418	-12,87754316	-222,9516912	-487,1685275	-223,9975	748,6278398	-743,64	=	4,99
87	18,7017143	-211,4104017	-216,8709636	51,97790052	-15,67131065	6,436254185	-6,055268365	-12,60287563	-101,7195975	-42,42493524	0,45202418	-6,944140281	-64,83084971	-76,38984661	-18,9402216	749,2319931	-743,64	=	5,60
88	24,4034932	-294,9416984	-231,5901772	77,52964674	-17,89218136	5,624709013	-9,656543771	-15,3839149	-95,04094631	-30,34144132	0,77070687	-9,062644624	-64,76301799	-58,41036276	-11,0853197	748,6248009	-743,64	=	4,99
89	22,129348	-282,5679678	-260,9682274	78,4738008	-21,30104567	7,876230927	-10,32639485	-19,34971763	-140,6045142	-52,79654084	0,87073574	-12,04294893	-101,2245192	-107,381458	-23,9700183	748,855981	-743,64	=	5,22
90	22,129348	-282,5679678	-277,2165668	78,4738008	-22,62728613	8,887539238	-10,32639485	-20,55446498	-158,6581385	-63,28490089	0,87073574	-12,79276404	-114,2217508	-128,7134502	-30,5207039	749,135283	-743,64	=	5,50
91	18,9140127	-243,63234	-207,2189758	68,25487017	-17,06238588	5,810147475	-9,060551501	-15,63545114	-104,6320967	-36,18270338	0,77070687	-9,816701342	-75,98858113	-74,23723503	-15,2612721	748,7461348	-743,64	=	5,11
92	18,9140127	-243,63234	-154,1614855	68,25487017	-12,69363843	3,215732268	-9,060551501	-11,63206394	-57,9105455	-14,89841623	0,77070687	-7,30317895	-42,0572685	-30,56756749	-4,67494161	748,6883675	-743,64	=	5,05
93	17,0399782	-232,2727388	-203,0694014	68,86124909	-17,69427091	6,193437084	-9,673281615	-17,15857734	-118,0286526	-41,95417587	0,87073574	-11,40025026	-90,70867085	-91,09067445	-19,2483952	748,7348322	-743,64	=	5,10
94	38,4984121	-363,3267724	-450,0663703	74,57605968	-27,15126844	13,46548795	-7,253101338	-18,22905287	-177,6655365	-89,47956229	0,45202418	-8,385376943	-94,53441097	-134,5079492	-40,2718401	749,7274709	-743,64	=	6,09
95	24,1429099	-256,039811	-276,1043526	59,0571971	-18,71759795	8,081066481	-6,454467886	-14,12171351	-119,8155199	-52,53156376	0,45202418	-7,299770107	-71,64124237	-88,73761054	-23,1285202	748,9925904	-743,64	=	5,36
96	30,6972415	-306,5771632	-356,0329886	66,59286505	-22,72948873	10,56800269	-6,853901817	-16,14914427	-147,5570207	-69,67096304	0,45202418	-7,861290884	-83,08688465	-110,8311349	-31,1090379	749,1299251	-743,64	=	5,49
97	24,1429099	-256,039811	-252,0337167	59,0571971	-17,08580736	6,733478248	-6,454467886	-12,89058977	-99,83523823	-39,95549376	0,45202418	-6,663379892	-59,69444111	-67,49380354	-16,0579244	749,026538	-743,64	=	5,39
98	24,1429099	-256,039811	-191,1491672	59,0571971	-12,95833705	3,873173876	-6,454467886	-9,776570891	-57,42637346	-17,43081929	0,45202418	-5,053686997	-34,33692682	-29,444569	-5,31305699	749,3017493	-743,64	=	5,67
99	19,5707501	-305,6017321	-211,0801851	103,7890652	-21,06952688	5,826385933	-16,70204575	-23,4057478	-127,1961895	-35,71965482	1,72227043	-17,81454617	-111,9836422	-88,84338764	-14,8317066	748,2130246	-743,64	=	4,58
100	32,4424547	-319,558643	-299,9794434	68,4596821	-18,88805911	7,098736728	-6,949306517	-13,23559872	-97,75622973	-37,31013903	0,45202418	-6,354543465	-54,28920122	-58,53737521	-13,281543	749,1196051	-743,64	=	5,48
101	24,1429099	-256,039811	-204,9543848	59,0571971	-13,89421694	4,452834991	-6,454467886	-10,48265657	-66,02083287	-21,48682329	0,45202418	-5,418675502	-39,47580824	-36,29607079	-7,02236971	749,288441	-743,64	=	5,65
102	26,5686155	-265,3627961	-194,730103	57,64459536	-12,43263496	3,652658338	-5,933342727	-8,833924664	-51,00428633	-15,21742715	0,39133968	-4,300596574	-28,72168627	-24,20927922	-4,29387383	749,5150985	-743,64	=	5,88
103	22,129348	-239,8511201	-231,1243386	56,54085	-16,01318761	6,177809788	-6,315463003	-12,34725231	-93,61257219	-36,67580089	0,45202418	-6,522999698	-57,20572044	-63,31728093	-14,7468936	749,1284545	-743,64	=	5,49
104	22,129348	-239,8511201	-221,8395732	56,54085	-15,36990318	5,69142755	-6,315463003	-11,85123644	-86,24240477	-32,43094661	0,45202418	-6,260956668	-52,70188376	-55,98894387	-12,5162407	749,1833748	-743,64	=	5,55
105	17,042679	-197,1826443	-222,4487643	49,61887996	-16,4520415	7,430771683	-5,916263482	-13,54160107	-120,1963422	-55,13077024	0,45202418	-7,636682573	-78,40688869	-101,6001615	-27,7032085	749,2230275	-743,64	=	5,59
106	12,8856432	-159,8802665	-214,8484776	43,14505976	-17,04040924	9,167908027	-5,516829552	-15,04139723	-159,032367	-86,8889277	0,45202418	-9,096636594	-111,2516429	-171,7207264	-55,7743471	749,2227632	-743,64	=	5,59
107	17,042679	-197,1826443	-219,4500677	49,61887996	-16,23026153	7,231782577	-5,916263482	-13,35905498	-116,9775968	-52,93113723	0,45202418	-7,533737097	-76,30722566	-97,54647121	-26,239343	749,2298845	-743,64	=	5,59
108	[illegible]	[illegible]	[illegible]	[illegible]	[illegible]	[illegible]	[illegible]	[illegible]	[illegible]	[illegible]	[illegible]	[illegible]	[illegible]	[illegible]	[illegible]	[illegible]	-743,64	=	5,51
